# PRDM16 acts as a therapeutic downstream target of TGF-**β** signaling in chronic kidney disease

**DOI:** 10.1172/jci.insight.191458

**Published:** 2025-07-29

**Authors:** Qian Yuan, Ben Tang, Yuting Zhu, Chao Wan, Yaru Xie, Yajuan Xie, Cheng Wan, Hua Su, Youhua Liu, Chun Zhang

**Affiliations:** 1 Department of Nephrology and; 2 Cancer Center, Union Hospital, Tongji Medical College, Huazhong University of Science and Technology, Wuhan, China; 3 State Key Laboratory of Organ Failure Research, National Clinical Research Center of Kidney Disease, Guangdong Provincial Institute of Nephrology, and Division of Nephrology, Nanfang Hospital, Southern Medical University, Guangzhou, China.

**Keywords:** Metabolism, Nephrology, Chronic kidney disease, Drug screens, Fibrosis

## Abstract

Transforming growth factor β (TGF-β) signaling is the master modulator of renal fibrosis. However, targeting drugs have failed to prevent the progression of chronic kidney disease (CKD) in clinical trials due to the extensive biological regulation of TGF-β signaling. It is necessary to investigate the precise downstream mechanisms of TGF-β signaling that regulate renal fibrosis. In this study, we found that PR-domain containing 16 (PRDM16) expression in human renal tubular epithelial cells was markedly reduced by TGF-β. Mechanistically, activated Smad3 induced by TGF-β interacted with the cofactor H-Ras and bound to the promoter of PRDM16, downregulating its transcription. Tubular-specific knockout of *Prdm16* promoted renal fibrosis in models of unilateral ureteral occlusion (UUO) and unilateral ischemia-reperfusion injury (UIRI) by exacerbating mitochondrial dysfunction. In vitro, PRDM16 blocked TGF-β–induced mitochondrial injury and lipid deposition by upregulating peroxisome proliferator-activated receptor γ coactivator-1α (PGC-1α). Delivery of the exogenous PRDM16 gene preserved renal function and ameliorated the progression of renal fibrosis by protecting mitochondrial function. We report PRDM16 as a potential downstream target of TGF-β signaling that attenuates renal fibrosis by safeguarding tubular mitochondrial function.

## Abstract 2

PRDM16 protects kidney function by counteracting TGF-β-induced mitochondrial damage, offering a potential therapeutic target for preventing renal fibrosis in chronic kidney disease.

## Graphical abstract

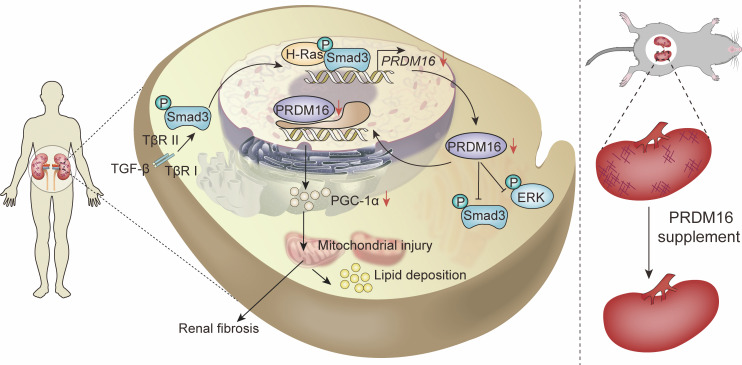

## Introduction

Chronic kidney disease (CKD) has become a worldwide public health problem with a high prevalence and high mortality (1). Renal fibrosis is a common pathology of CKD and a key accelerator of the progression of CKD to end-stage renal disease (ESRD) (2–4). Thus, attenuating renal fibrosis is an effective approach for decreasing the number of patients with ESRD. However, therapies for ameliorating renal fibrosis are limited.

Transforming growth factor β (TGF-β) signaling is a developmental pathway that regulates multiple normal physiological processes such as cell growth and differentiation, autophagy, and the immune response (5). In the canonical TGF-β signaling cascade, TGF-β1 binds to the TGF-β receptor dimer and activates Smad2 and Smad3. Phosphorylated Smad2/3 translocates into the nucleus to regulate downstream targets. TGF-β signaling has been extensively studied in the pathogenesis of organ fibrosis (6). It is a master regulator of renal fibrosis that modulates fibroblast activation and proliferation, tubular injury through partial epithelial-to-mesenchymal transition (EMT), and energy metabolism disorders, endothelial-to-myofibroblast transition, and macrophage-to-myofibroblast transition (7). Due to their strong role in renal fibrosis, TGF-β signaling–targeted drugs have been widely investigated and shown to display encouraging antifibrotic effects in preclinical animal studies (8). Pirfenidone (NCT00063583), LY2382770 (NCT01113801), and Fresolimumab (NCT01665391) are the most promising potential candidates for treating patients with renal fibrosis (9). However, the results from clinical trials of these drugs are disappointing (10, 11). We noticed that these drugs blunted the interaction between TGF-β and the TGF-β receptor, blocking the entire TGF-β signaling cascade, which governs numerous biological responses in addition to fibrosis. Thus, exploring the specific downstream factors of TGF-β signaling that regulate renal fibrosis is necessary.

The PRDF1 and RIZ1 homology domain (PRDM) protein family contains 17 members who share the common conserved N-terminal PR domain and a variable number of zinc fingers (ZNF) (12). PRDM proteins mediate histone methylation due to the similarity of the PR domain to the SET domain, which resides on histone lysine methyltransferases (HMTs). The ZNF domain of PRDM binds to DNA and regulates the transcription of target genes (13). PRDM proteins participate in the regulation of various diseases, including lymphoma, leukemia, cardiomyopathy, and metabolic disorders (12). However, the role of the PRDM family in renal diseases has not been fully elucidated.

We measured the expression of the PRDM family in human tubular epithelial cells exposed to TGF-β1. We found that PRDM16 expression decreased the most after TGF-β1 treatment. Phosphorylated Smad3 after treatment of TGF-β decreased the transcription of PRDM16 by binding to its promoter. H-Ras acted as transcriptional coactivator of p-Smad3 to regulate the expression of PRDM16. The tubular-specific knockout of PRDM16 aggravated renal fibrosis and mitochondrial dysfunction in unilateral ureteral occlusion (UUO) and unilateral ischemia-reperfusion injury (UIRI) mice. Peroxisome Proliferator-Activated Receptor γ Coactivator-1α (PGC-1α) mediated the regulatory effect of PRDM16 on tubular mitochondrial homeostasis. Furthermore, we demonstrated the antifibrotic role of PRDM16 in UIRI mice and folic acid nephropathy mice with an overexpression lentivirus. These results suggest that PRDM16 is a precise downstream target of TGF-β signaling that mediates renal fibrosis by improving tubular mitochondrial function.

## Results

### PRDM16 is decreased in the injured kidneys of mice and humans.

**Figure 1:**
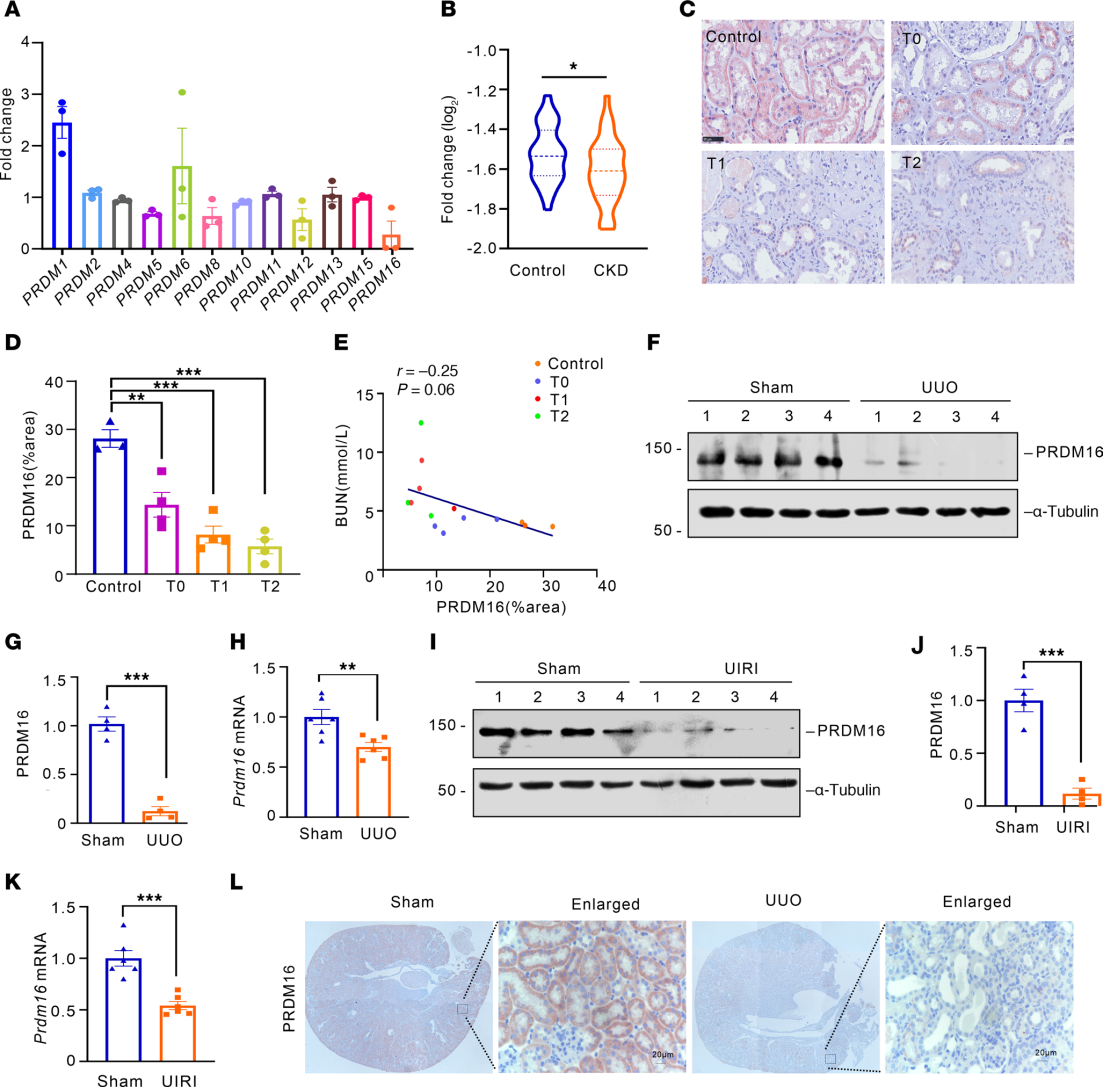
PRDM16 was decreased in the injured kidneys of mice and humans. (**A**) Fold change of PRDM-family in HK-2 cells exposed to TGF-β for 24 hours (*n =* 3); (**B**) The expression of PRDM16 in normal controls and patients with CKD from the nephroseq database; (**C** and **D**) Representative immunohistochemical images of PRDM16 (**C**) and quantification of the PRDM16-positive area (**D**) in renal biopsy samples from IgA nephropathy patients (T0–T2) and control individuals (*n =* 3–4). Scale bar: 50 μm; (**E**) Correlation between the serum urea nitrogen level and the quantification of the PRDM16-positive area in all participants (*n =* 14); (**F** and **G**) Representative Western blotting (**F**) (*n =* 4) and quantification of PRDM16 (**G**) in kidneys from the sham and UUO groups; (**H**) Relative mRNA level of *PRDM16* in UUO mice (*n =* 6); (**I** and **J**) Representative Western blotting (**I**) (*n =* 4) and quantification of PRDM16 (**J**) in the kidneys of UIRI mice; (**K**) Relative mRNA level of *PRDM16* in sham and UIRI kidneys (*n =* 6); (**L**) Representative immunohistochemical staining images of PRDM16 in the kidneys of sham and UUO mice. Scale bar: 20 μm. Data are mean ± SEM. *P < 0.05, **P < 0.01, ***P < 0.001.Two-tailed Student’s unpaired *t* test analysis (**B**, **D**, **G**, **H**, **J**, and **K**), Spearman’s correlations (**E**).

We conducted RNA-seq with human renal tubular epithelial cells (HK-2 cells) exposed to TGF-β (Supplemental Figure 1, A and B; supplemental material available online with this article; https://doi.org/10.1172/jci.insight.191458DS1). The results showed that the expression of *PRDM16* decreased the most among the PRDM family members after TGF-β treatment (Figure 1A). Then, we analyzed the RNA-seq data derived from the tubulointerstitial compartment of kidney biopsies of patients with CKD in the Nephroseq database (14). The results showed that PRDM16 expression was decreased significantly in patients with CKD (Figure 1B). Kidney paraffin biopsies from IgA patients with different stages of tubular atrophy/interstitial fibrosis (T) according to the Oxford classification were collected, and IHC staining revealed that the expression of PRDM16 decreased with the progression of fibrosis (Figure 1, C and D). The expression of PRDM16 was negatively correlated with blood urea nitrogen (Figure 1E). The results of Western blotting and qRT-PCR showed that the levels of PRDM16 were reduced in the UUO kidney and the UIRI kidney (Figure 1, F–K). IHC staining showed that PRDM16 was mainly expressed in healthy tubular epithelial cells, but not in UUO tubules (Figure 1L). Together, these findings indicated that PRDM16 was diminished markedly in the fibrotic kidneys of humans and mice.

### TGF-β transcriptionally downregulates PRDM16 in an H-Ras/p-Smad3–dependent manner.

**Figure 2:**
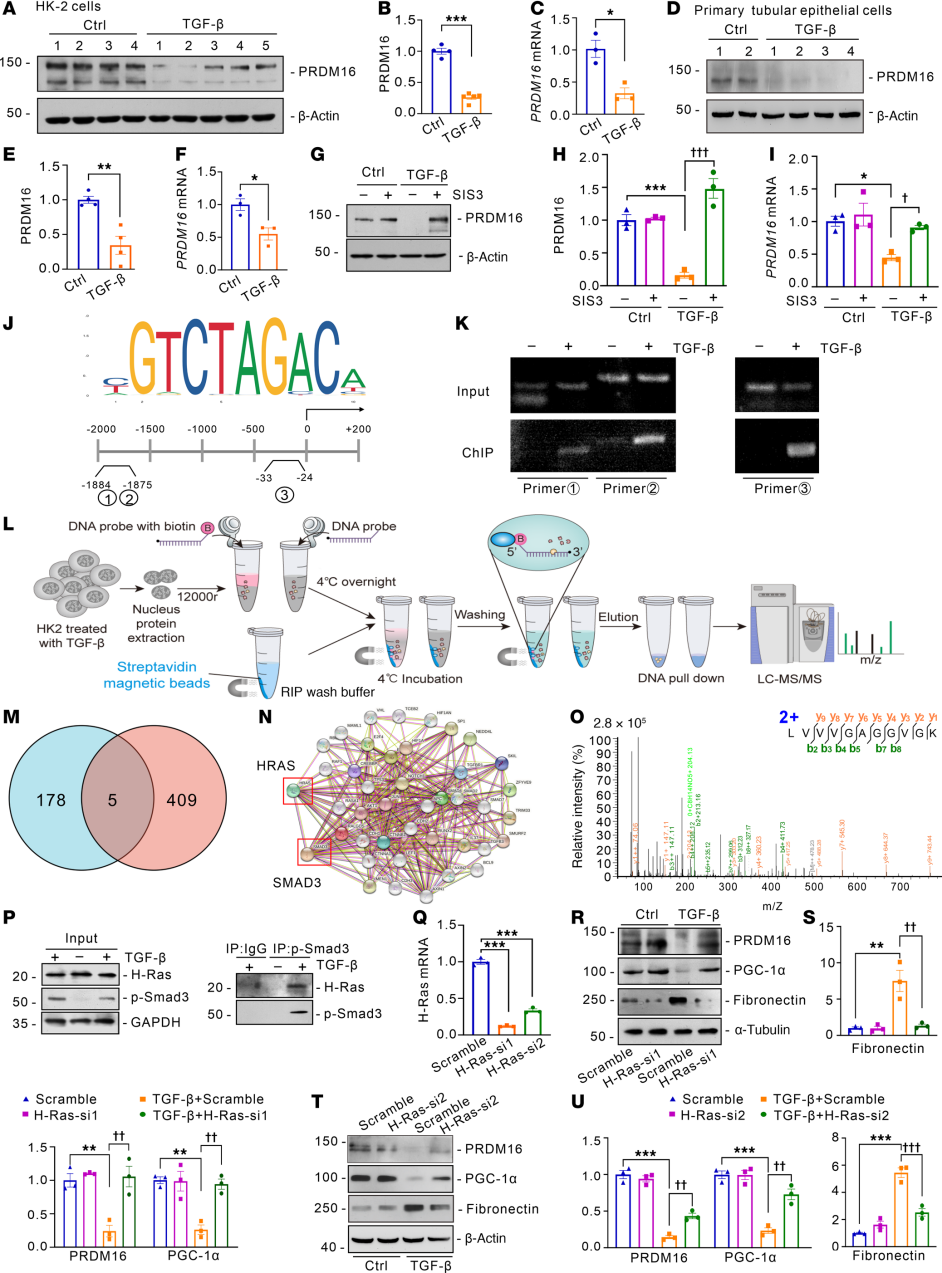
TGF-β transcriptionally downregulated PRDM16 in an H-Ras/p-Smad3–dependent way. (**A** and **B**) Representative Western blotting (**A**) (*n =* 4 or 5) and quantification of PRDM16 (**B**) in HK-2 cells exposed to 5 ng/ml TGF-β for 24 hours; (**C**) Relative mRNA level of *PRDM16* in HK-2 cells (*n =* 3); (**D** and **E**) Western blotting (**D**) (*n =* 4) and quantification of PRDM16 (**E**) in primary mouse tubular epithelial cells exposed to TGF-β for 24 hours; (**F**) *PRDM16* mRNA levels in primary tubular epithelial cells (*n =* 3); (**G**–**I**) The protein lysates of HK-2 cells provided with TGF-β and SIS3 (5 μmol/L) for 24 hours were used. Western blotting and quantification (**G** and **H**), and mRNA level (**I**) (*n =* 3). were shown; (**J**) The predicted motif of the Smad3 DNA-binding domain from jaspar.genereg.net and primers were designed to target the latent binding site of Smad3 at the PRDM16 promoter. (**K**) Representative ChIP-PCR images of HK-2 cells exposed to 5 ng/ml TGF-β or vehicle for 45 minutes, the experiment was repeated 3 times. (**L**) Schematic diagram of DNA pull-down; (**M**) The differential proteins in DNA pull-down complex shared 5 proteins with the Wound healing GO set (GO: 0042060); (**N**) Protein-protein interaction network of H-Ras and Smad3 according to the STRING database; (**O**) The mass spectrum of H-Ras; (**P**) Coimmunoprecipitation of H-Ras and p-Smad3 in HK-2 cells exposed to TGF-β for 45 minutes; (**Q**) Relative mRNA levels of *H-Ras* in HK-2 cells (*n =* 3) transfected with H-Ras siRNA1 (H-Ras-si1), H-Ras siRNA2 (H-Ras-si2), or Scramble; (**R**–**U**) HK-2 cells provided with TGF-β, and H-Ras siRNA1 (**R** and **S**) or siRNA2 (**T** and **U**) transfection for 24 hours. Representative Western blotting (*n =* 3) and quantification of PRDM16, PGC-1α, and fibronectin were shown. Data are mean ± SEM. *P < 0.05, **P < 0.01, ***P < 0.001; ^††^*P* < 0.01, ^†††^*P* < 0.001. Asterisks indicate comparison to sham or control group. Crosses indicate comparison to TGF-β or UUO/UIRI/FA groups. Two-tailed Student’s unpaired *t* test analysis (**B**, **C**, **E**, **F**, and **Q**), One-way ANOVA followed by Tukey’s post test (**H**, **I**, **S**, and **U**).

We investigated the mechanism by which TGF-β regulated PRDM16. TGF-β greatly decreased the protein and mRNA levels of PRDM16 in HK-2 cells (Figure 2, A–C). We observed results consistent with those obtained with cultured primary tubular epithelial cells from mice (Figure 2, D–F). Specific inhibitor of Smad3 (SIS3), which specifically inhibits the phosphorylation of Smad3, was used to demonstrate the role of Smad3 in the regulation of PRDM16 (15). As expected, p-Smad3 inhibition reversed the decrease in the protein and mRNA levels of PRDM16 caused by TGF-β treatment (Figure 2, G–I). Does p-Smad3 downregulate PRDM16 by binding to its promoter? By analyzing the JASPAR database, we found multiple Smad3 DNA-binding elements within the PRDM16 promoter (Figure 2J). We designed 3 primers to recognize these binding elements. ChIP showed PRDM16 promoter could be detected with three primers in the pull-down complex precipitated with anti-p-Smad3 antibodies (Figure 2K). These results indicated that p-Smad3 downregulated PRDM16 by binding to its promoter. Although Smad3 acts as a transcription factor, its affinity for DNA is weak. Thus, the transcription coactivator is necessary for the high affinity and high specificity of Smad3 to the promotor (16). To further investigate the transcription cofactor of Smad3 that regulates the transcription of PRDM16, we conducted DNA pull-down assay. As shown in Figure 2L, we provided HK-2 cells with TGF-β and extracted the nuclear proteins. The nuclear proteins were incubated with streptavidin magnetic beads, and biotin-labeled DNA probes that bound specifically to the promoter of PRDM16. After repeated washing, the pulled down proteins were analyzed by LC-MS/MS proteomics. A total of 183 proteins differed from those in the negative DNA probe control group, and they shared only 5 proteins with the “wound healing” data set (GO:0042060) (Figure 2M). The 5 proteins were H-Ras, Vinculin, Talin-1, CASK, and Carmil2. H-Ras was the only protein that was predicted to interact with SMAD3 by the STRING database (Figure 2N). The mass spectrum of H-Ras was shown in Figure 2O. H-Ras was found in the immunocomplexes precipitated by the anti-p-Smad3 antibodies during coimmunoprecipitation (Figure 2P). H-Ras is a member of the Ras family of GTPase. We knocked down H-Ras with two siRNAs to validate the effect of H-Ras on PRDM16 expression and tubular injury (Figure 2Q). Mitochondrial dysfunction and partial EMT are defining features of tubular injury. Among these, the downregulation of PGC-1α is a key driver of mitochondrial dysfunction (17). Partial EMT, marked by the acquisition of mesenchymal traits in tubular epithelial cells, promotes the secretion of profibrotic mediators that activate interstitial fibroblasts, leading to excessive extracellular matrix (ECM) deposition and the progression of renal fibrosis (18). Western blotting showed that TGF-β treatment decreased the expression of PRDM16 and PGC-1α and increased the level of fibronectin; however, H-Ras knockdown blocked these changes (Figure 2, R–U). Collectively, Smad3 activated by TGF-β downregulates the transcription of PRDM16 with the assistance of H-Ras.

### The tubular-specific knockout of Prdm16 aggravates ischemia-reperfusion induced renal interstitial fibrosis and tubular mitochondrial dysfunction.

**Figure 3:**
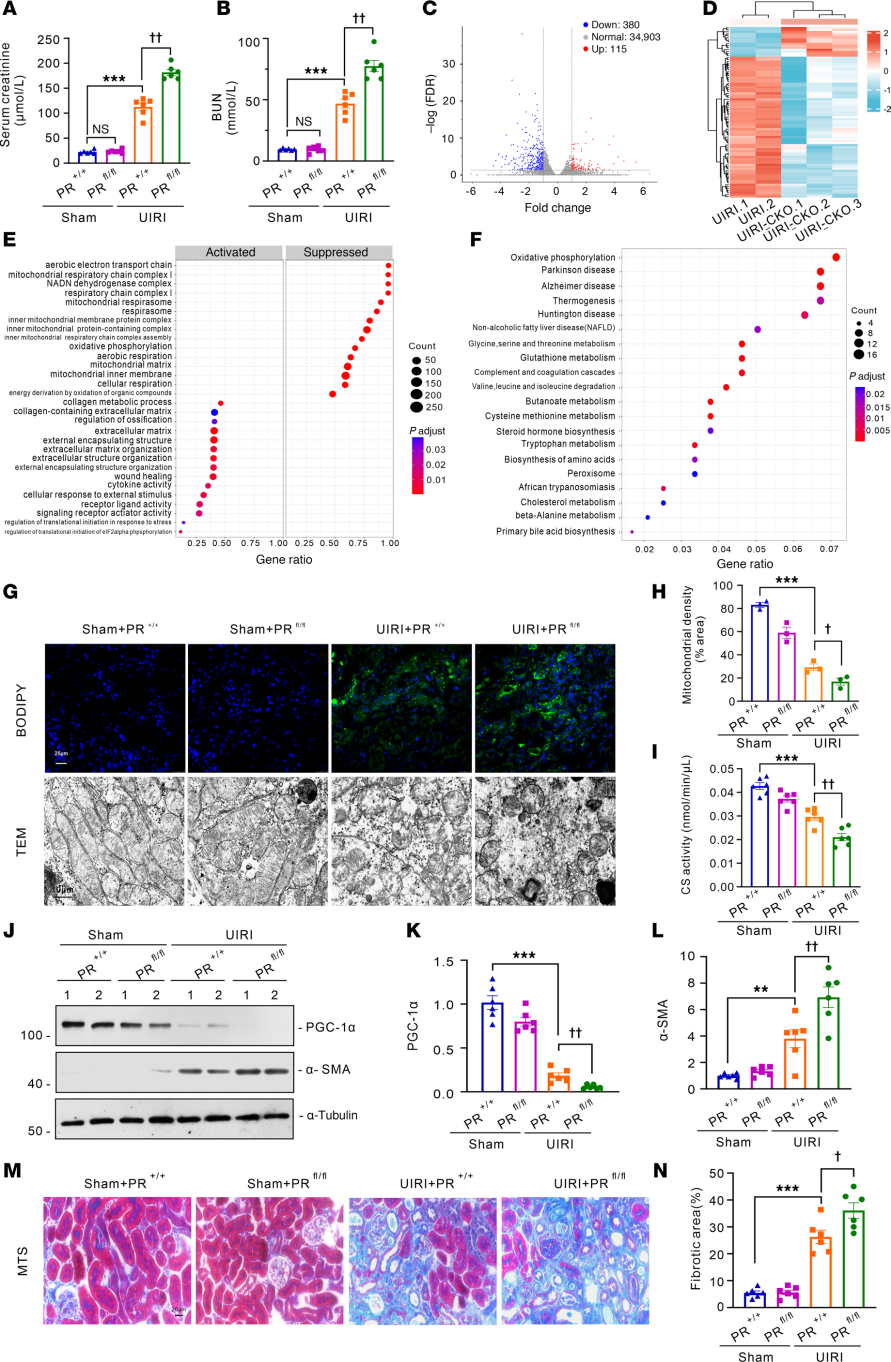
Tubular-specific knockout *Prdm16* aggravated ischemia-reperfusion–induced renal interstitial fibrosis and tubular mitochondrial dysfunction. (**A** and **B**) Serum creatinine (Scr) (**A**) and blood urea nitrogen (BUN) (**B**) levels of the *PRDM16^fl/fl^* (PR^+/+^) and *Ksp-Cre*/*PRDM16^fl/fl^* (PR^fl/fl^) mice subjected to UIRI or sham surgery (*n =* 6); (**C**) Volcano plot of the RNA-seq data; (**D**) Heatmap of the RNA-seq data; (**E**) GSEA of RNA-seq data; (**F**) KEGG analysis of RNA-seq data; (**G**) Representative images of Boron-dipyrromethene (BODIPY) staining, Scale bar: 25μm. Representative images of transmission electron microscopy (TEM). Scale bar: 10 μm; (**H**) Quantification of mitochondrial density (*n =* 3); (**I**) Citrate synthase activity of each group (*n =* 6); (**J**–**L**) Representative Western blotting (**J**) (*n =* 6) and quantification of PGC-1α (**K**) and α-SMA (**L**) in kidney lysates from PR^+/+^ and PR^fl/fl^ mice; (**M** and **N**) Representative images of Masson trichrome (MTS) staining (**M**) and quantification of the fibrotic area (**N**) of each group (*n =* 6). Data are mean ± SEM.**P < 0.01, ***P < 0.001; ^†^*P* < 0.05,^††^*P* < 0.01. Asterisks indicate comparison to sham or control group. Crosses indicate comparison to TGF-β or UUO/UIRI/FA groups. One-way ANOVA followed by Tukey’s post test (**A**, **B**, **H**, **I**, **K**, **L**, and **N**).

To demonstrate the role of PRDM16 in kidney fibrosis, we generated tubular-specific *Prdm16* knockout mice by hybridizing *Prdm16^fl/fl^* mice and *Ksp-Cre* mice (19). Genotyping and immunofluorescence staining revealed that only PRDM16 expressed by tubular epithelial cells was knocked out (Supplemental Figure 2, A–C). Then, we performed UIRI surgery on the transgenic mice. Serum creatinine (Scr) and urea nitrogen (BUN) levels were greater in *Ksp-Cre/Prdm16 ^fl/fl^* mice than in *Prdm16^fl/fl^* mice after injury (Figure 3, A and B). To determine the influence of PRDM16 knockout on the expression profile, we conducted RNA-seq of kidneys from *Ksp-Cre/Prdm16^fl/fl^* mice and *Prdm16^fl/fl^* mice with UIRI. Volcano plots and heatmaps showed that the gene expression was quite different between the 2 groups (Figure 3, C and D). As demonstrated by GSEA, compared with those of the *Prdm16^fl/fl^* kidney, the top 15 suppressed GO sets of the *Ksp-Cre/Prdm16^fl/fl^* mice were all related to mitochondria, and 7 of the top 15 activated GO sets were related to the extracellular matrix (Figure 3E). KEGG analysis showed that oxidative phosphorylation was the most common pathway (Figure 3F). These results indicated that PRDM16 deficiency likely promoted mitochondrial structure and functional disorders. Then, we performed experiments to confirm this hypothesis. Mitochondrial injury impairs fatty acid oxidation and results in lipid deposition. BODIPY staining showed that lipid deposition was much more severe after PRDM16 knockout (Figure 3G). As shown by TEM images, mitochondrial cristae were impaired in UIRI transgenic mice. PRDM16 knockout exacerbated mitochondrial structure damage and the decrease in mitochondrial density (Figure 3, G and H). Mitochondrial function, as revealed by citrate synthase activity, was worse in *Ksp-Cre/Prdm16^fl/fl^* UIRI mice than in *Prdm16^fl/fl^* UIRI mice (Figure 3I). Western blot analysis demonstrated a significant decrease in the expression of PGC-1α in mice subjected to UIRI. PRDM16 knockout further downregulated the expression of PGC-1α (Figure 3, J and K). Then, renal interstitial fibrosis was evaluated. Western blotting of α-SMA and Masson trichrome staining (MTS) revealed that the kidneys of *Ksp-Cre/Prdm16^fl/fl^* mice exhibited increased myofibroblast accumulation and ECM deposition after UIRI surgery (Figure 3, J and L–N).

### The tubular-specific knockout of Prdm16 promotes tubular mitochondrial injury and interstitial fibrosis in UUO mice.

**Figure 4:**
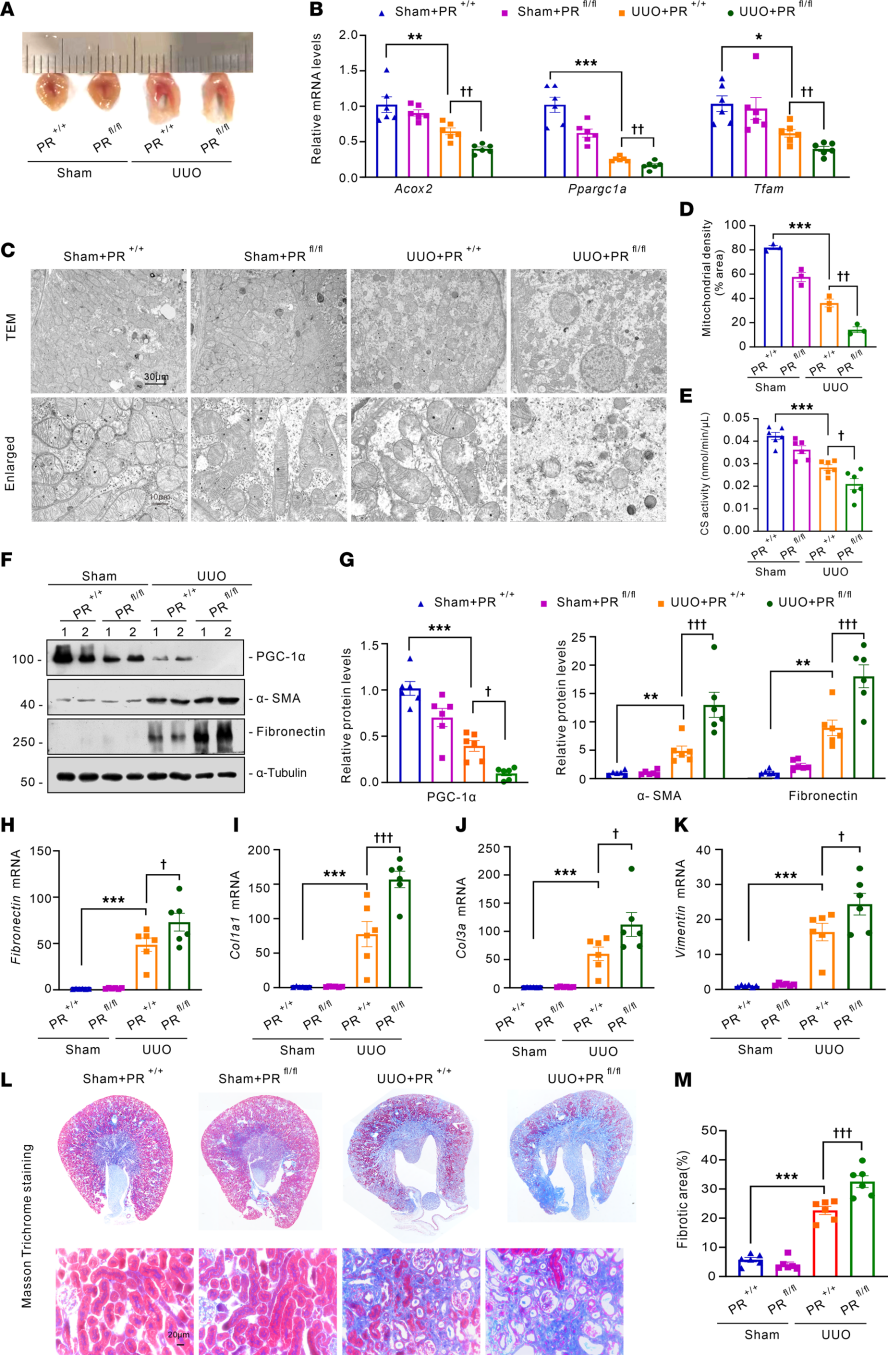
Tubular-specific knockout *Prdm16* promoted tubular mitochondrial injury and interstitial fibrosis of UUO mice. (**A**) Representative kidney cross-sections from each group; (**B**) Relative mRNA levels of *Acox2*, *Ppargc1a*, and *Tfam* in the renal cortex of PR^+/+^and PR^fl/fl^ mice (*n =* 6); (**C**) Representative transmission electron microscopy (TEM) images showing the structure and number of mitochondria in tubular epithelial cells. Scale bar: 30 μm, 10 μm; (**D**) Quantification of mitochondrial density (*n =* 3); (**E**) Citrate synthase activity of each group (*n =* 6); (**F** and **G**) Representative Western blotting (**F**) (*n =* 6) and quantification (**G**) of PGC-1α, α- SMA, and Fibronectin (*n =* 6); (**H**–**K**) Relative mRNA levels of *fibronectin*, *Col1a1*, *Col3a*, and *Vimentin* genes (*n =* 6); (**L** and **M**) Representative images of Masson trichrome staining (**L**) and quantification of the fibrotic area (**M**) of each group (*n =* 6). Data are mean ± SEM. *P < 0.05, **P < 0.01, ***P < 0.001; ^†^*P* < 0.05, ^††^*P* < 0.01, ^†††^*P* < 0.001. Asterisks indicate comparison to sham or control group. Crosses indicate comparison to TGF-β or UUO/UIRI/FA groups.One-way ANOVA followed by Tukey’s post test (**B**, **D**, **E**, **G**–**K**, and **M**).

We utilized the UUO animal model, another widely recognized model for studying renal fibrosis to assess the impact of PRDM16 on renal fibrosis. The renal cortex and medulla were thinner in *Ksp-Cre/Prdm16 ^fl/fl^* UUO mice than in *Prdm16 ^fl/fl^* UUO mice (Figure 4A). The expression of mitochondrial-related genes such as *Acox2, Ppargc1a,* and *Tfam* decreased more in the kidneys of *Ksp-Cre/Prdm16^fl/fl^* UUO mice than in those of *Prdm16^fl/fl^* UUO mice (Figure 4B). Transmission electron microscopy (TEM) revealed that mitochondrial loss and cristae disappearance were aggravated after *Prdm16* knockout (Figure 4, C and D). Citrate synthase activity was lower in the *Prdm16*-knockout mice (Figure 4E). The expression of PGC-1α was inhibited in the UUO kidney, and knockdown of *Prdm16* exacerbated this decrease (Figure 4, F and G). Then, we further assessed renal fibrosis in the UUO model. Moreover, the protein levels of fibronectin and α-SMA and the mRNA levels of *Fibronectin*, *Col1al*, *Col3a*, and *Vimentin* were increased by UUO surgery and were exaggerated by *Prdm16* knockout (Figure 4, F–K). Collagen deposition, as revealed by Masson’s trichrome staining, was more severe in *Ksp-Cre/Prdm16^fl/fl^* UUO mice than in *Prdm16^fl/fl^* UUO mice (Figure 4, L and M). These results suggest that the loss of PRDM16 promoted mitochondrial dysfunction and the progression of renal fibrosis.

### PRDM16 inhibits TGF-β induced tubular mitochondrial dysfunction and TGF-β signaling.

**Figure 5:**
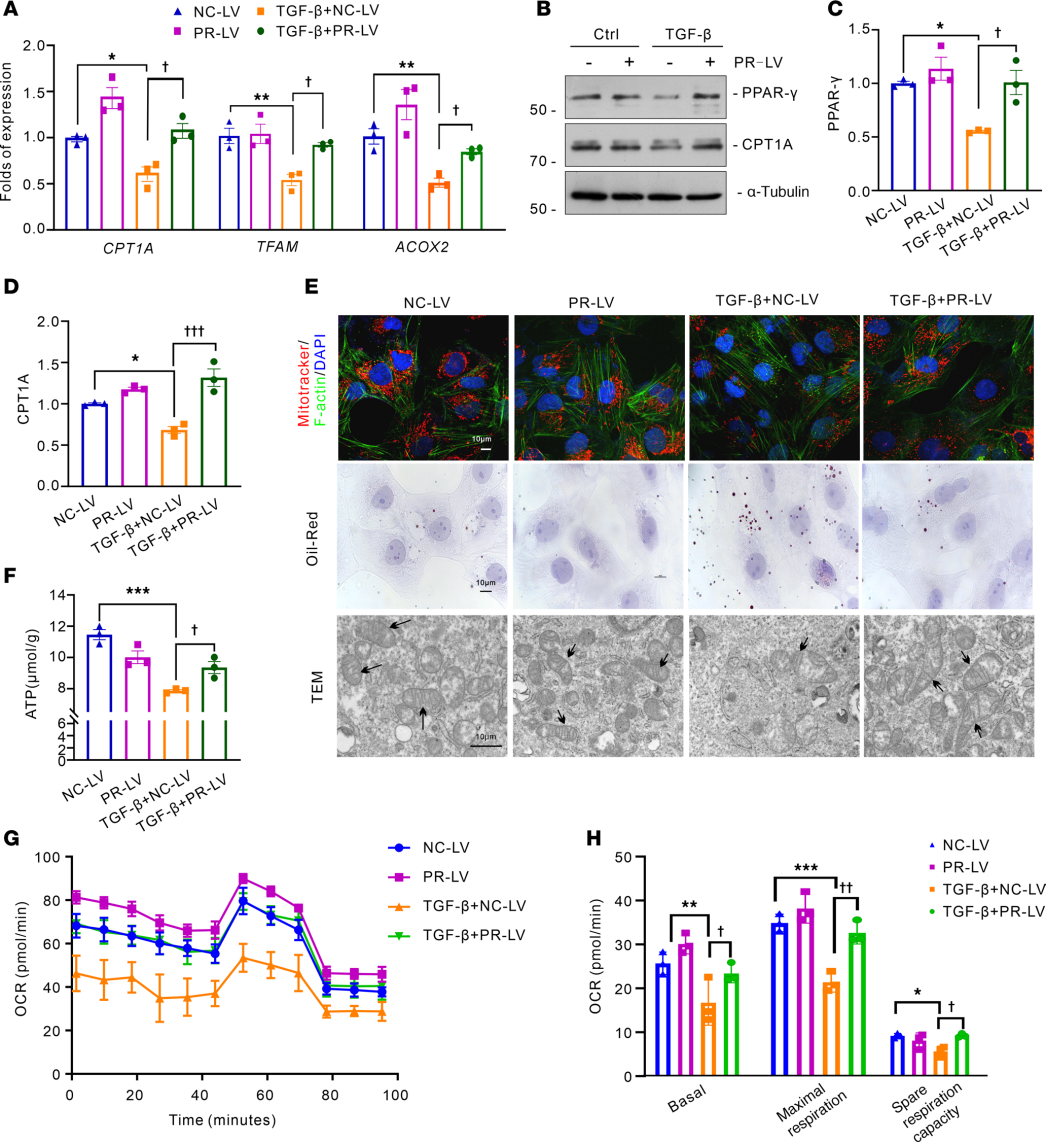
PRDM16 inhibited TGF-β induced tubular mitochondrial dysfunction and TGF-β signaling. (**A**) Relative mRNA levels of *CPT1A*, *TFAM,* and *ACOX2* in control lentivirus (NC-LV)-transfected HK-2 cells and PRDM16-overexpressing lentivirus (PR-LV)-transfected HK-2 cells exposed to 5 ng/ml transforming growth factor-β (TGF-β) for 24 hours (*n =* 3); (**B**–**D**) Representative Western blotting (**B**) (*n =* 3) and quantification of PPAR-γ (**C**) and CPT1A (**D**) in stably transfected HK-2 cells after TGF-β treatment for 24 hours; (**E**) Representative images of MitoTracker staining, Oil Red O staining and TEM images. In the first row of the figures, red indicates MitoTracker staining, green indicates phalloidin staining, and blue indicates DAPI. Scale bar: 10 μm. The arrows indicate the mitochondrial cristae; (**F**) Measurement of adenosine triphosphate (ATP) levels in stably transfected HK-2 cells exposed to TGF-β (*n =* 3); (**G** and **H**) The oxygen consumption rate was measured with a Seahorse bioanalyzer. Data are mean ± SEM. *P < 0.05, **P < 0.01, ***P < 0.001; ^†^*P* < 0.05, ^††^*P* < 0.01, ^†††^*P* < 0.001. Asterisks indicate comparison to sham or control group. Crosses indicate comparison to TGF-β or UUO/UIRI/FA groups. One-way ANOVA followed by Tukey’s post test (**A**, **C**, **D**, **F**, and **H**).

To investigate the renoprotective effect of PRDM16, we screened and obtained PRDM16-overexpressing HK-2 cell lines with lentivirus (Supplemental Figure 3). PRDM16-overexpressing HK-2 cells were exposed to vehicle or TGF-β. PRDM16 overexpression blocked the decrease in *TFAM*, *CPT1A,* and *ACOX2* caused by TGF-β (Figure 5A). Western blotting showed that PRDM16 overexpression blocked the reduction in PPAR-γ and CPT1A induced by TGF-β (Figure 5, B–D). As shown in Figure 5E, PRDM16 overexpression inhibited the mitochondrial loss, mitochondrial swelling, and lipid accumulation caused by TGF-β treatment. In addition, ATP production was rescued by PRDM16 overexpression (Figure 5F). We measure the oxygen consumption rate (OCR) with a Seahorse bioanalyzer. The basal OCR, maximal respiration, and spare respiration capacity were impaired after TGF-β treatment, and PRDM16 overexpression counteracted the mitochondrial dysfunction caused by TGF-β (Figure 5, G and H). PRDM16 also mitigated the partial EMT process in HK-2 cells induced by TGF-β (Supplemental Figure 4). The effect of PRDM16 on TGF-β signaling was detected. Western blotting revealed that PRDM16 overexpression decreased the phosphorylation of Smad3 and ERK in HK-2 cells exposed to TGF-β (Supplemental Figure 5, A–C). The nuclear translocation of p-Smad3 after TGF-β incubation was inhibited by PRDM16 (Supplemental Figure 5D).

### PRDM16 rescues tubular mitochondrial function by upregulating PGC-1α.

**Figure 6:**
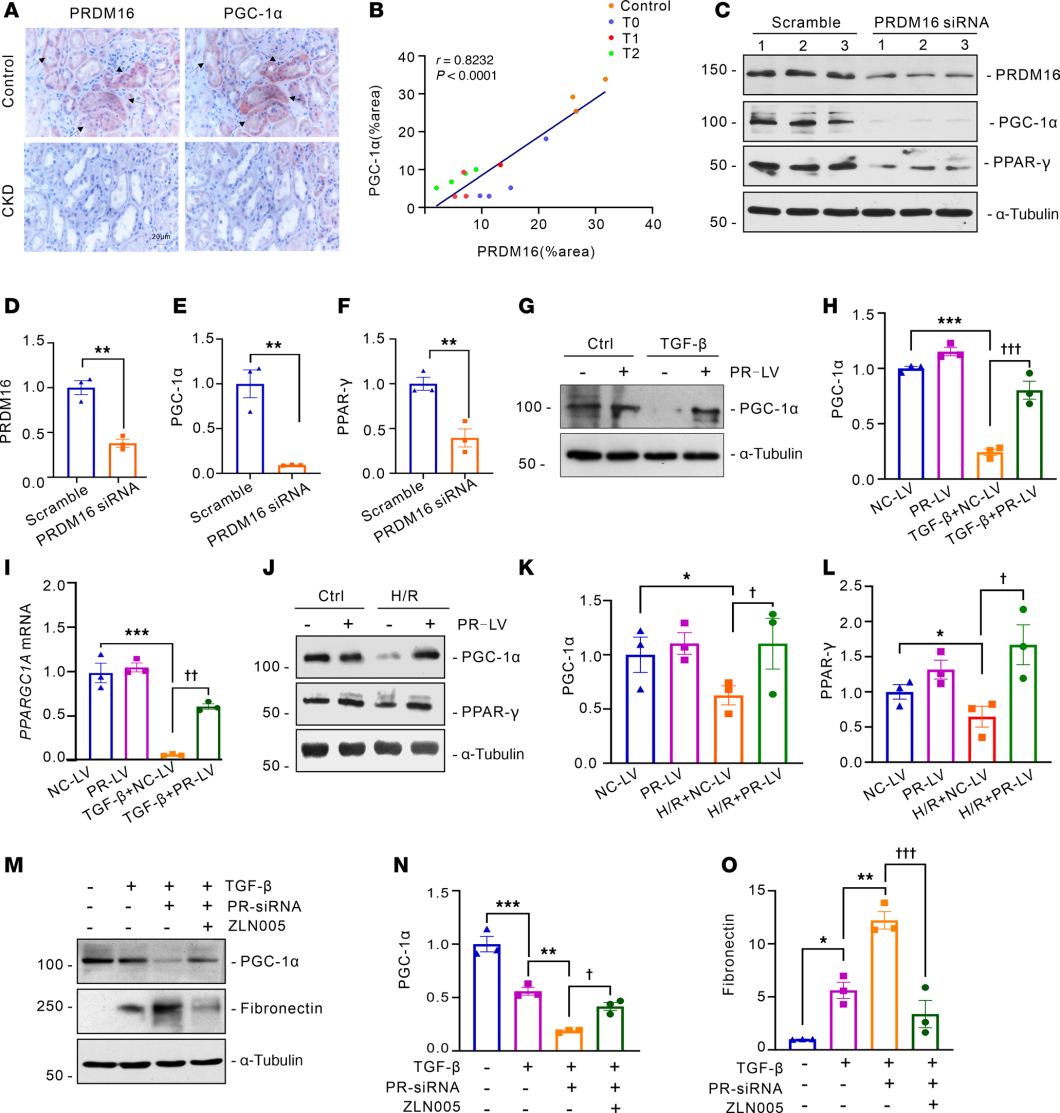
PRDM16 rescued tubular mitochondrial function via upregulating PGC-1α. (**A**) Representative IHC images of the costaining of PRDM16 and PGC-1α in serial renal biopsy sections from patients with CKD. Scale bar: 20 μm. The black arrow indicates positive costaining tubules; (**B**) Correlation between the quantification of the positively stained area of PRDM16 and PGC-1α (*n =* 15); (**C**–**F**) HK-2 cells were transfected with PRDM16 siRNA or scrambled siRNA for 24 hours. Representative Western blotting (**C**) (*n =* 3) and quantification of PRDM16 (**D**), PGC-1α (**E**), and PPAR-γ (**F**); (**G** and **H**) Representative Western blotting (*n =* 3) and quantification of PGC-1α in stably transfected HK-2 cells after TGF-β treatment for 24 hours; (**I**) Relative mRNA level of *PPARGC1A* in stably transfected HK-2 cells after TGF-β treatment for 24 hours. (*n =* 3); (**J**–**L**) Stably transfected HK-2 cells were subjected to hypoxia for 24 hours and reperfusion for 2 hours. Representative Western blotting (**J**) (*n =* 3) and quantification of PGC-1α (**K**) and PPAR-γ (**L**); (**M**–**O**) HK-2 cells were provided with TGF-β, PRDM16 siRNA (PR-siRNA), Scramble, or ZLN005 for 24 hours according to the group assignment. Representative Western blotting (**M**) and quantification of PGC-1α (**N**) and fibronectin (**O**) were shown. Data are mean ± SEM. *P < 0.05, **P < 0.01, ***P < 0.001; ^†^*P* < 0.05, ^††^*P* < 0.01, ^†††^*P* < 0.001. Asterisks indicate comparison to sham or control group. Crosses indicate comparison to TGF-β or UUO/UIRI/FA groups. Spearman’s correlations (**B**). Two-tailed Student’s unpaired *t* test analysis (**D**–**F**), One-way ANOVA followed by Tukey’s post test (**H**, **I**, **K**, **L**, **N**, and **O**).

PGC-1α plays a crucial role in the regulation of mitochondrial biosynthesis (20). Then, we investigated the regulatory effect of PRDM16 on PGC-1α. IHC staining of serial sections from patients with CKD showed that PRDM16 costained with PGC-1α (Figure 6A), and the expression of PRDM16 was positively correlated with PGC-1α (Figure 6B). We used 2 different siRNAs to knock down PRDM16 in vitro (Figure 6, C and D, and Supplemental Figure 6, A–E). The protein levels of PGC-1α and PPAR-γ were downregulated after PRDM16 knockdown (Figure 6, C–F, and Supplemental Figure 6, D–G). PRDM16 overexpression blocked the decrease of PGC-1α induced by TGF-β (Figure 6, G–I), as well as the downregulation of PGC-1α and PPAR-γ caused by hypoxia and reoxygenation injury (Figure 6, J–L). The compound ZLN005 functions as a potent agonist of PGC-1α by effectively enhancing its transcription through direct interaction with the promoter region (21). ZLN005 alleviated the increase of fibronectin, mitochondrial loss, and lipid accumulation induced by TGF-β in vitro (Supplemental Figure 7). The rescue experiments showed that ZLN005 blunted the decrease of PGC-1α and the increase of fibronectin caused by PRDM16 knockdown (Figure 6, M–O). These data indicate that PRDM16 prevents mitochondrial dysfunction in vitro by upregulating PGC-1α.

### Prdm16 gene delivery via lentivirus attenuates mitochondrial dysfunction and interstitial fibrosis in vivo.

**Figure 7:**
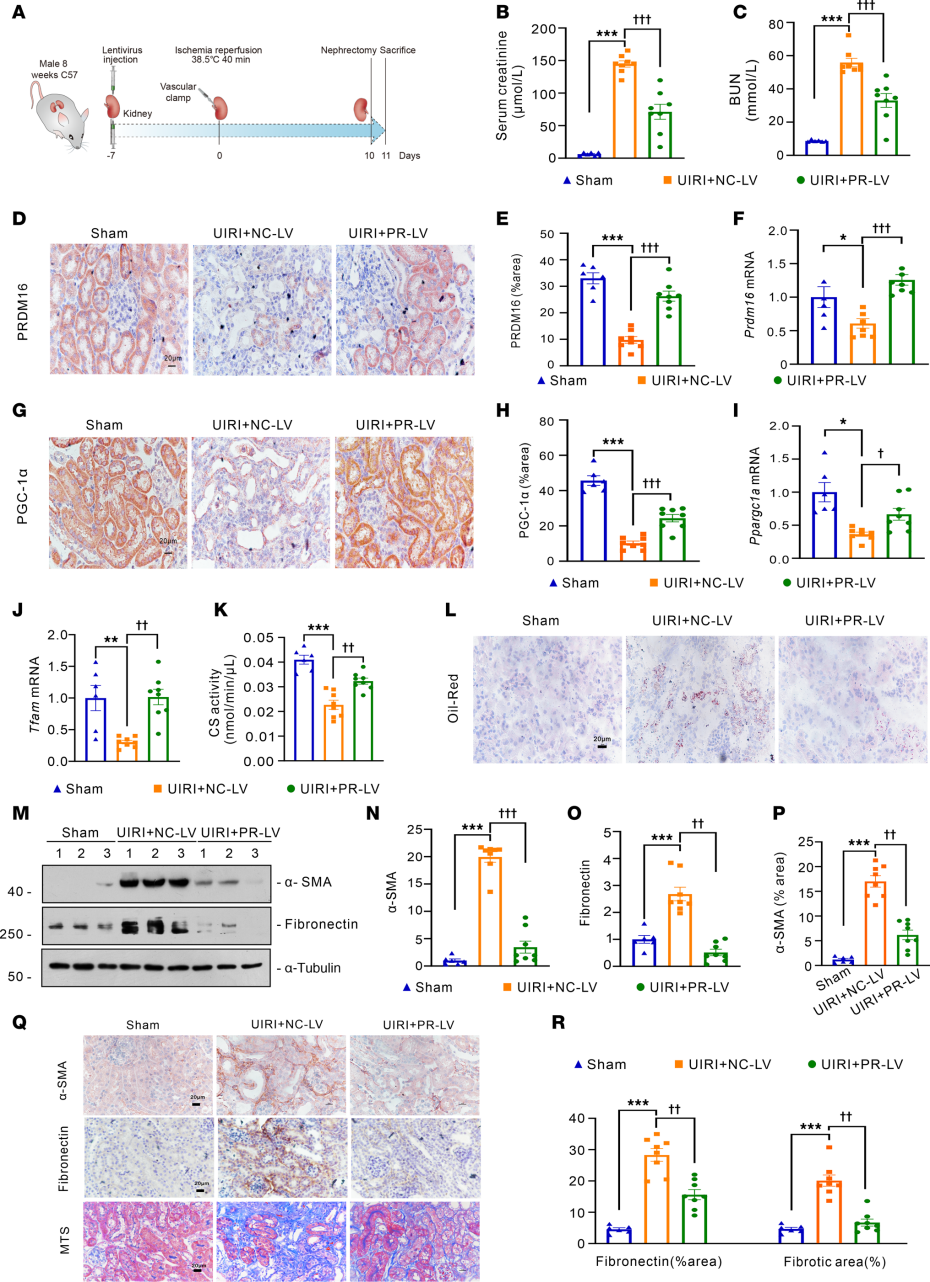
Renal injection with PRDM16 overexpressing lentivirus attenuated mitochondrial dysfunction and interstitial fibrosis in the UIRI model. (**A**) Schematic diagram of the UIRI model with lentivirus injection; (**B** and **C**) Serum creatinine and BUN levels in each group (*n =* 6 or 8); (**D**) Representative immunohistochemical staining images of PRDM16. Scale bar: 20 μm; (**E**) Ratio of the PRDM16-positive area to the total area (*n =* 6 or 8); (**F**) Relative mRNA level of *PRDM16* in the renal cortex of each group (*n =* 6 or 8); (**G**) Representative immunohistochemical staining images of PGC-1α. Scale bar: 20 μm; (**H**) Ratio of the PGC-1α–positive area to the total area (*n =* 6 or 8); (**I**) Relative mRNA level of *Ppargc1a* in the renal cortex of each group (*n =* 6 or 8); (**J**) Relative mRNA level of *TFAM* in the renal cortex of each group (*n =* 6 or 8); (**K**) Citrate synthase activity of each group (*n =* 6 or 8); (**L**) Representative images of Oil Red O staining. Scale bar: 20 μm; (**M**–**O**) Representative Western blotting (**M**) (*n =* 6 or 8) and quantification of α-SMA (**N**) and Fibronectin (**O**) with kidney pole cortex lysates; (**P**–**R**) Representative images (**Q**) of IHC staining of α- SMA and Fibronectin, and Masson-trichrome staining and quantification of the α-SMA-positive area (**P**), the Fibronectin-positive area and the fibrotic area (**R**) (*n =* 6 or 8). Scale bar: 20 μm. Data are mean ± SEM. *P < 0.05, **P < 0.01, ***P < 0.001; ^†^*P* < 0.05, ^††^*P* < 0.01, ^†††^*P* < 0.001. Asterisks indicate comparison to sham or control group. Crosses indicate comparison to TGF-β or UUO/UIRI/FA groups. One-way ANOVA followed by Tukey’s post test (**B**, **C**, **E**, **F**, **H**–**K**, **N**–**P**, and **R**).

To explore the renoprotective effect of PRDM16 in vivo, we sought to deliver the exogenous PRDM16 gene with lentivirus and then performed ischemia-reperfusion surgery on the injected kidney. Ten days later, we removed the other kidney and euthanized the mice after 24 hours (Figure 7A). Renal functions reflected by Scr and BUN were increased in UIRI mice, but PRDM16 overexpression reduced their levels (Figure 7, B and C). As illustrated in Figure 7, D and E, the PRDM16 protein was overexpressed in the tubular epithelial cells of the UIRI mice. qRT-PCR analysis of *Prdm16* mRNA expression showed similar results (Figure 7F). The expression of the downstream target of PRDM16, PCG-1α, was restored due to PRDM16 overexpression, as demonstrated by IHC staining (Figure 7, G and H) and qRT-PCR (Figure 7I). The mRNA level of *Tfam* was increased in the kidneys of UIRI mice after PRDM16 overexpression (Figure 7J). Mitochondrial activity revealed by citrate synthase activity was damaged in UIRI mice but was significantly attenuated after PRDM16 overexpression (Figure 7K). Mitochondrial repair facilitates fatty acid oxidation (FAO) and subsequently protects tubular cells from lipid deposition. Lipid accumulation, as revealed by Oil Red O staining, was alleviated by PRDM16 overexpression (Figure 7L). We have shown the mitochondrial protective role of PRDM16. Then, we further assessed the effects of PRDM16 on renal interstitial fibrosis. Western blotting showed that the overexpression of PRDM16 inhibited the renal expression of fibronectin and α-SMA in UIRI mice (Figure 7, M–O). IHC staining of α-SMA and fibronectin showed consistent results (Figure 7, P–R). PRDM16 overexpression also attenuated collagen deposition and fibrotic lesions in the kidney, as shown by Masson’s trichrome staining (Figure 7, Q and R).

**Figure 8:**
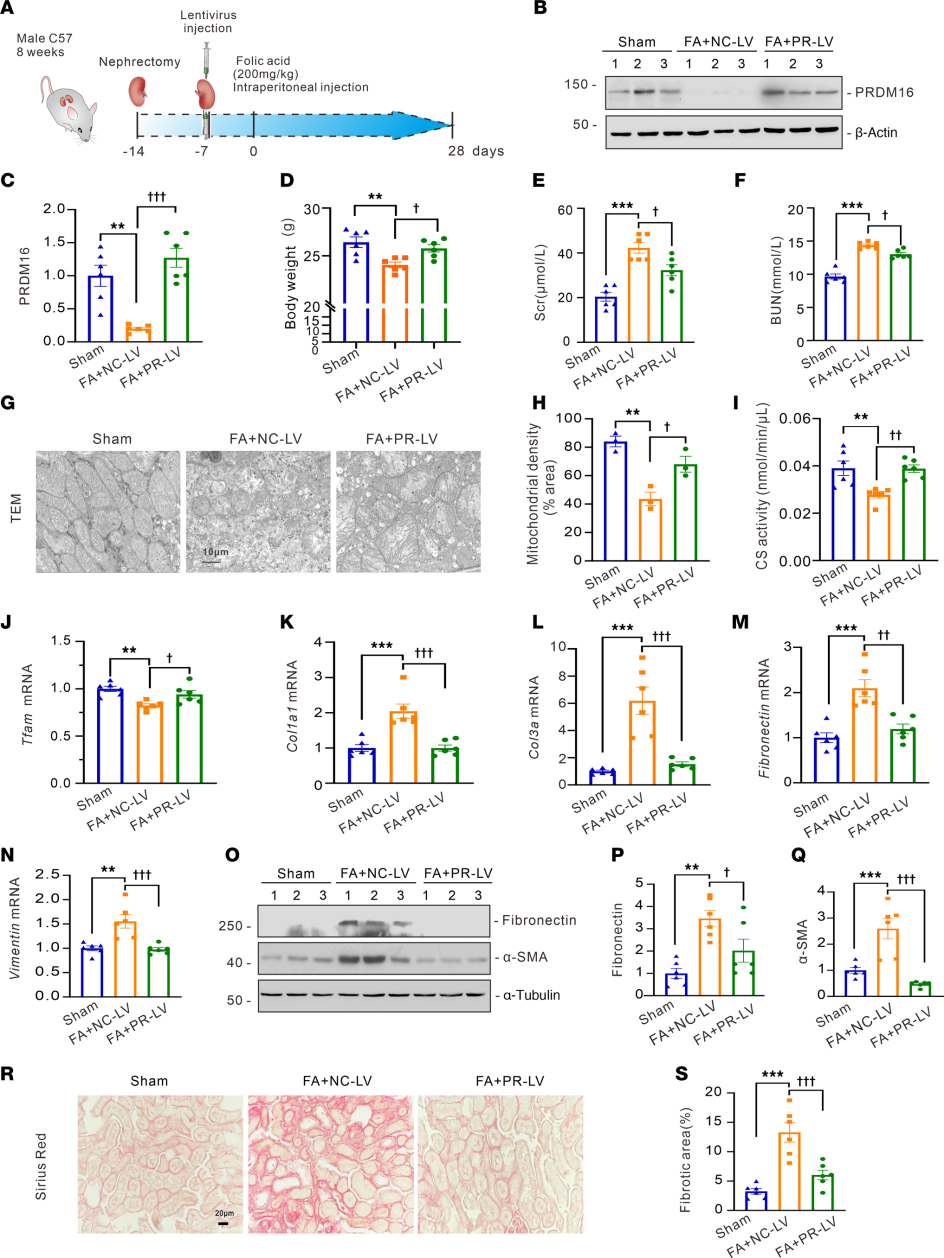
Renal PRDM16 lentivirus injection attenuated folic acid–induced mitochondrial damage and interstitial fibrosis. (**A**) Schematic diagram of the folic acid nephropathy model with lentivirus injection; (**B** and **C**) Representative Western blotting (**B**) (*n =* 6), and quantification of PRDM16 (**C**) in kidney cortex lysates; (**D**) Body weight of each group (*n =* 6); (**E** and **F**) Serum creatinine and BUN levels in each group (*n =* 6); (**G** and **H**) Representative images of TEM and quantification of mitochondrial density (*n =* 3). Scale bar: 10 μm; (**I**) Citrate synthase activity of each group (*n =* 6); (**J**–**N**) Relative mRNA levels of *TFAM, Col1a1, Col3a*, *Fibronectin*, and *Vimentin* in renal cortex of each group (*n =* 6); (**O**–**Q**) Representative Western blotting (**O**) (*n =* 6) and quantification of Fibronectin (**P**), α-SMA (**Q**) and kidney pole cortex lysates; (**R** and **S**) Representative images (**R**) of Sirius Red staining. Scale bar: 20 μm. (**S**) Quantification of the fibrotic area (*n =* 6). Data are mean ± SEM. **P < 0.01, ***P < 0.001; ^†^*P* < 0.05, ^††^*P* < 0.01, ^†††^*P* < 0.001. Asterisks indicate comparison to sham or control group. Crosses indicate comparison to TGF-β or UUO/UIRI/FA groups. One-way ANOVA followed by Tukey’s post test (**C**–**F**, **H**–**N**, **P**, **Q**, and **S**).

Furthermore, a folic acid–induced renal fibrosis model was established to verify the renoprotective role of PRDM16. As illustrated in Figure 8A, after right nephrectomy and left renal lentivirus injection, the mice were treated with folic acid (200 mg/kg) intraperitoneally. Western Blotting results showed that the PRDM16-overexpressing lentivirus was successfully transfected into the kidneys of folic acid–treated mice (Figure 8, B and C). Then, we analyzed the levels of body weight, Scr, and BUN. The results showed that PRDM16 overexpression restored the decrease of the body weight and the increase of Scr and BUN of folic acid mice (Figure 8, D–F). TEM was used to observe the mitochondrial structure and density. As shown in Figure 8, G and H, mitochondrial cristae and number were decreased in folic acid–treated mice, and PRDM16 protected mitochondria from damage. Citrate synthase activity and the mRNA level of *Tfam* were restored by PRDM16 overexpression in folic acid–treated mice (Figure 8, I and J). We also demonstrated that renal fibrosis was attenuated by PRDM16 overexpression, as the mRNA levels of *Col1a1*, *Col3a*, *Fibronectin*, and *Vimentin* (Figure 8, K–N), the protein levels of Fibronectin and α-SMA (Figure 8, O–Q), and collagen deposition (Figure 8, R and S) decreased. Thus, these data revealed that delivery of PRDM16 gene is effective in protecting kidneys from chronic injury.

## Discussion

In this study, we demonstrated that PRDM16 was a potential downstream target of the TGF-β signaling pathway. PRDM16 attenuated the progression of renal fibrosis by protecting tubular mitochondrial function. TGF-β transcriptionally decreased the expression of PRDM16 in the fibrotic kidney in an H-Ras/Smad3–dependent way. Delivery of the exogenous PRDM16 gene attenuated renal fibrosis and preserved tubular mitochondrial function by upregulating PGC-1α.

To identify potential downstream pathways of TGF-β signaling, the expression of PRDM members were assessed in human tubular epithelial cells exposed to TGF-β. Among these genes, PRDM16 exhibited the greatest decrease. While PRDM1 exhibited significant upregulation in a PRDM isoforms screening experiment, our prioritization of PRDM16 was driven by the following interrelated biological and methodological rationales. PRDM16 is a critical regulator of mitochondrial biogenesis and cellular metabolic adaptation, which plays a key role in maintaining the physiological function of tubular cells (22). In contrast, PRDM1 (also known as BLIMP1) is predominantly associated with immune cell differentiation (23), which aligns poorly with our focus on tubular epithelial cell injury mechanisms. It has been reported that PRDM16 knockout in T cells facilitates adoptive immunotherapies for cancer (24). Whether PRDM16 plays a hostile role in renal fibrosis needs further study. Then, we investigated the regulatory effect of TGF-β on PRDM16. According to the data shown above, PRDM16 was downregulated by TGF-β/Smad3 at the transcriptional level. Hariom Yadav et al. showed that the expression of PRDM16 in fat tissue increased after Smad3 knockout (25). However, this study did not investigate the mechanism by which TGF-β/Smad3 regulates PRDM16 expression. We detected H-Ras from the pull-down complex of the PRDM16 promoter, indicating that Smad3 specifically downregulated the expression of PRDM16 with the help of H-Ras.

We further demonstrated that tubular-specific knockout of PRDM16 aggravated the progression of renal fibrosis. It should be noted that our findings are limited to male participants. Published papers suggest sexual dimorphism in renal fibrosis progression, with male kidneys demonstrating heightened inflammatory and fibrotic response compared with females (26). Future investigations will incorporate both sexes to evaluate potential sex-specific effects. According to the analysis of the RNA-seq data, mitochondrial dysfunction was the most obvious change in the kidney after PRDM16 knockout. Mitochondrial dysfunction blocks fatty acid oxidation (FAO), which is the primary energy source for tubular epithelial cells, causing lipid accumulation, oxidative stress, partial EMT, and even cell death (19, 27). Injured renal tubular epithelial cells secrete multiple cytokines, including TGF-β, Wnt, and IL-1β, to recruit immune cells and activate myofibroblasts, resulting in the progression of renal fibrosis (28). Furthermore, we showed that PRDM16 preserved tubular mitochondrial function by upregulating the expression of PGC-1α. Previous research has found that the PGC-1α level decreases in patients with CKD and in mice, with TGF-β playing a vital role in this decline (4). The downregulation of PGC-1α leads to mitochondrial dysfunction, increased ROS production, and intracellular lipid deposition (29, 30). However, overexpression of PGC-1α rescued these changes induced by Notch overexpression (31). The level of PGC-1α exhibits a positive correlation with glomerular filtration rate, while conversely, it demonstrates a negative correlation with renal fibrosis (32, 33). The possible mechanism underlying the regulatory effect of PRDM16 on PGC-1α in adipocytes has been discussed. The activity of the PGC-1α promoter was enhanced by PRDM16 through indirect DNA binding. The PGC-1α protein, which is a feedback transcriptional coactivator of its own gene, is required for the regulatory effect of PRDM16 on PGC-1α (34–36). In addition, PRDM16 inhibited the phosphorylation of Smad3, which transcriptionally downregulated the expression of PGC-1α (25, 37). PRDM16 functions as a histone methyltransferase through its conserved SET domain. Previous studies have demonstrated its ability to mediate the methylation of H3K4, H3K9, and H3K36 (38, 39). Notably, the demethylation of H3K9 at the Pgc1 gene promoter has been shown to repress transcriptional activation (40). Therefore, the reduced PGC-1α expression observed in PRDM16-deficient conditions may be attributed to the demethylation of H3K9. We also showed that PRDM16 not only was downstream of TGF-β signaling but also inhibited the activation of Smad3 and ERK induced by TGF-β. It has been reported that PRDM16 represses the transcriptional activity of TGF-β signaling and tumor cell proliferation by binding to p-Smad3 and suppressing the interaction of p-Smad3 with DNA (37). However, how PRDM16 inhibits the activation of Smad3 and ERK is unclear. The potential mechanism, as inferred from published studies, is that PRDM16 inhibits the transcription of TGF-β by modifying the methylation levels of H3K4 and H3K9 (41). In the present study, PRDM16 also influenced lipid deposition in tubular epithelial cells. The underlying mechanism involves PRDM16-mediated mitochondrial repair, which enhances FAO (10, 42). Another possible mechanism is that PRDM16 activates several FAO-related genes directly, including *CPT1A*, *UCP-1*, and *PPAR-α* (43–45).

Finally, we showed that lentivirus-mediated delivery of the PRDM16 gene protected kidneys from UIRI and folic acid injury by preserving mitochondrial function. Similarly, Xu et al. recently reported that the induction of PRDM16 was the reason for low tubular interstitial fibrosis in the early stage of diabetic kidney disease (46). Previous studies have indicated that the disappointing results of TGF-β inhibitors in clinical trials are largely related to the inhibition of TGF-β in regulatory T cells, which exacerbates autoimmune disease (7). It has been reported that mitochondrial oxidative damage occurs in regulatory T cells of individuals with autoimmunity. The scavenging of mtROS attenuates autoimmunity by preventing regulatory T cell death (47). PRDM16 has a powerful effect on protecting mitochondrial function and improving oxidative phosphorylation. It is suspected that PRDM16 may restrict autoimmune disease by protecting regulatory T cells. However, PRDM16 has diverse roles across different organs. In the blood system, overexpression of PRDM16 promotes leukemia development​, which highlights the importance of targeted delivery (48). To accurately target renal cells while minimizing systemic exposure and potential side effects, we propose utilizing high-affinity peptides targeting the protein expressed by injured renal tubular epithelial cells (e.g., Kim-1) for tissue-specific interventions. These peptides will be conjugated to the surface of nanomaterials that encapsulate PRDM16, enabling precise and efficient targeted delivery to the kidney.

In this study, we clarified the antirenal fibrosis role of tubular PRDM16. And PRDM16 is a key regulator of adipose metabolism, including adipocyte thermogenesis, adipogenic differentiation of myoblasts, and visceral-to-subcutaneous adipocyte conversion (12, 13). The kidneys are surrounded by a fat pad, which is termed perirenal adipose tissue (PRAT). Due to the adjacent anatomical location, PRAT thickness increase was associated with a higher incidence of CKD, while total body fat (TBF), subcutaneous adipose tissue (SAT), or visceral adipose tissue (VAT) were not. The underlying mechanism of PRAT in the regulation of renal damage has not been elucidated completely. The possible reasons are the direct compression of PRAT on the kidney, the disorder of serum lipid and glucose metabolism, and the release of proinflammatory adipokines and chemokines (49, 50). Whether PRDM16 expressed by perirenal adipocytes plays an important role in PRAT browning, other metabolic changes, and renal fibrosis remains to be further studied.

In summary, this study identified a potential target, PRDM16, for attenuating renal fibrosis. To apply these findings in the clinic, we plan to overexpress PRDM16 in vivo by designing mRNA drugs and screening agonists or mimic peptides in the future. We illustrated that TGF-β decreased the transcription of PRDM16 in an H-Ras/Smad3-dependent manner. The compounds that block the interaction between H-Ras and Smad3 could also be potential options for treating patients with CKD.

## Methods

### Sex as a biological variable.

Our mice study exclusively examined male mice. It is unknown whether the findings are relevant for female mice. In our study of renal biopsy samples, both male and female human kidney samples were used; however, the biological variable examined in the experiment was the degree of fibrosis, with sex not being considered a biological variable.

### Materials and human renal biopsy samples.

Fresh biopsies were dehydrated and paraffin embedded. The control samples were healthy kidney tissues adjacent to kidney tumors. The extent of tubular atrophy and interstitial fibrosis, which are < 25%, 25%–50%, and > 50% of the cortical area are defined as T0, T1, and T2, respectively, on the basis of the Oxford classification of IgA. The clinical characteristics and serum sample analysis results are listed in Supplemental Table 1.

### Mouse study.

Wild-type and transgenic mice were housed under controlled environmental conditions with ad libitum access to water and a standard mouse chow diet. Cages containing standard corncob bedding were changed twice weekly. Littermate control mice were used for all in vivo experiments. The experimenter conducting animal experiments was aware of the group allocation, while the data collection and analysis personnel remained blinded. The number of mice utilized in each experiment is specified in the figure legends as per published papers.

### Generation of tubular epithelial cell-specific knockout mice.

*Prdm16^fl/fl^* mice (C57BL/6J;129) were generated by Shanghai Southern Model Biotechnology Development Co. Ltd. In brief, the process involved obtaining Cas9 mRNA and gRNA through in vitro transcription, constructing a donor vector for In-Fusion cloning, and microinjecting these components into fertilized eggs of C57BL/6J mice to produce F0 generation mice. Positive F0-generation mice were identified using PCR amplification and sequencing. Then, F0 generation mice were mated with C57BL/6J mice to obtain positive F1 generation mice. *Prdm16* exon9 was flanked by loxP sequences. The *Prdm16^fl/fl^* mice were cross mated with *Ksp-Cre* mice (C57BL/6J;129). And then *Prdm16^fl/fl^* mice and *Ksp-Cre/Prdm16^flox/+^* mice were cross mated. The breeding of transgenic mice was conducted by Shulaibao Biotechnology Co., Ltd (Shulaibao, Wuhan, China).

### Genotyping of tubular-specific Prdm16 knockout mice.

Glomeruli and tubules isolation method was as described previously (51). Mice were anesthetized and injected with 20 ml prewarmed PBS from left ventricle, and then injected with 37°C prewarmed inactivated Dynabeads (8×10^7^ beads/mouse) in 20 ml PBS immediately. Kidneys were removed. Kidney cortex was separated and minced into 1 mm^3^ pieces, and digested with collagenase 2 buffer (1 mg/ml) at 37 °C for 15 minutes. The kidneys were digested and the glomeruli were isolated with dynabeads using a magnetic concentrator, while the remaining fluids were centrifuged to collect the tubules. DNA was extracted from glomeruli, tubules, heart, liver, spleen, and lung with Genomic DNA Extraction Kit (D0063, Beyotimes, Wuhan, China). In general, tissue particles within 10 mg were dissolved in 180 μl lysate and 20 μl proteinase K at 55°C overnight. After washing of absolute alcohol and scrubbing solution, DNA was collected. PCR with the following primers were used for transgenic mice genotyping. *Ksp-Cre* primer was used to identify the expression of *Ksp-Cre*. The floxP primer 1 whose products contained the 5’ loxP was used to identify whether loxP was inserted successfully. The products of floxP primer 2 contained exon 9 and two loxP. Exon 9 is a long exon that has more than 2,000 bp. Thus, the products of floxP primer 2 were less than 500 bp only if exon 9 was knocked out.

### UIRI and UUO model with transgenic mice.

Male *Prdm16^fl/fl^* mice and *Ksp-Cre/Prdm16^fl/fl^* mice aged 7–8 weeks were used. UUO and UIRI were performed as described in previous studies (52). To be specific, UUO model was that the left ureter was obstructed with 3–0 suture in the location paralleled to inferior pole of kidney. Mice were euthanized in 7 days. Left kidney was collected to do further experiments. In UIRI model, pedicle of left kidney was clipped with vascular clamp, and mice were put on constant temperature metal bath at 38.5°C for 35 minutes. At 10 days after UIRI, right kidney was removed. Mice were euthanized after 24 hours.

### UIRI model with PRDM16 lentivirus.

Male C57BL/6J mice at 7 weeks of age were procured from Vital River Laboratory Animal Technology Co. Ltd (Beijing, China). We first diluted lentivirus with ice-cold germfree PBS solution. Left kidney was exposed from the back of mice and injected with lentivirus equivalently from the upper pole and inferior pole of kidney. 5×10^6^ IU lentivirus in 100 μl solution was injected to per kidney. 8 mm insulin injectors were used to avoid bleeding. 7 days after lentivirus injection, ischemia reperfusion surgery as described above was conducted. Mice were put on constant temperature metal bath at 38.5°C for 40 minutes. The remaining steps were the same as the UIRI model of transgenic mice.

### Folic acid nephropathy model with PRDM16 lentivirus.

Male C57BL/6J mice at 7 weeks of age were procured from Vital River Laboratory Animal Technology Co. Ltd. Right kidney was removed 7 days before lentivirus injection. Negative control and PRDM16 Lentivirus were diluted by germfree PBS solution. Mice were anesthetized. Left kidney was injected with lentivirus. 7 days after lentivirus injection, one single intraperitoneal injection of folic acid (HY-16637, MCE, NJ, USA) at a dose of 200 mg/kg dissolved in 0.3M NaHCO3 was performed to induce folic acid nephropathy model. Mice were euthanized after 28 days of folic acid injection.

### Cell culture and treatments.

HK-2 cells were obtained from American Type Culture Collection (ATCC) and were cultured in cell incubator with 37°C, 5% carbon dioxide. DMEM/F12 (Gibco, NY, USA) medium with 10% fetal bovine serum (FBS) were used to culture HK-2 cells. For TGF-β treatment experiments, cells were seeded in 6 well plates and cultured in serum-free medium with TGF-β (5 ng/mL) after adherence (53). Cells were harvested in 24 hours for mitochondrial function detecting, and in 45 minutes for p-Smad3 and p-ERK detecting. In hypoxia and reperfusion experiments, hypoxia 24 hours and reperfusion 2 hours were used. SIS3(5 μmol, HY-13013, MCE, NJ, USA) and ZLN005 (5 μmol HY-17538, MCE, NJ, USA) were used.

### Primary tubular epithelial cells culture.

Mouse primary tubular epithelial cells were isolated and cultured following established procedures (52). Renal cortex was minced into 1 mm^3^ particles in ice-cold germfree PBS solution, and was digested by collagenase II (C6885, Sigma, MO, USA) at 37°C for 40 minutes and was mixed every 10 minutes. The tissues were filtered by 40 mesh sieve and 100 μm filter successively. Tubules on 100 μm filter were collected and resuspended in DMEM/F12 culture medium with 10% bovine calf serum, 50 U/ml penicillin, and 50 mg/ml streptomycin. After adhesion, primary tubular cells were exposed to TGF-β1(5 ng/ml) for 24 hours.

### Real time quantitative PCR.

The mRNA of kidney tissue or cells was extracted by classical procedures with trizol and trichloromethane. 1 μg mRNA was used to reverse transcription to cDNA with 2 step assays (R323-01, Vayzame, Nanjing, China). Quantitative reverse transcription PCR (qRT-PCR) was conducted on Step one plus system with the AceQ qPCR SYBR Green Master Mix (Q111, Vayzame, Nanjing, China). The reaction system (20 μl) was constituted by 10 μl SYBRmix, 0.4 μl forward primers, 0.4 μl reverse primers, 0.4 μl ROX dye1, 3.8 μl RNase free water and 5 μl cDNA (diluted for 10 times). Primers of targeted genes involved were listed in the Supplemental Table 2. The relative mRNA level of genes was calculated with 2-ΔΔCT and normalized with the housekeeping gene β-actin.

### Western blot analyses.

Western blotting protocols have been described previously (52), kidney cortex (54) or cultured cells were lysed in RIPA buffer (G2002, Servicebio) containing 1x protease inhibitor cocktail (G2006, Servicebio, Wuhan, China), 1x PMSF (ST506, Beyotime Biotechnology, Wuhan, China) for at least 10 mins on ice and centrifugated at 4°C for 15 mins 13,400 xg. The supernatant of centrifugation was collected and separated by SDS-PAGE and transferred onto 0.45 μm PVDF membranes. Proteins were incubated with primary antibodies overnight at 4°C and with secondary antibodies for 1 hour at room temperature. Antibodies involved are summarized in Supplemental Table 3. The housekeeping proteins α-tubulin, β-actin, and GAPDH were used as loading controls.

### Immunofluorescence staining.

The 2 μm frozen renal sections or cell coverslips were fixed with 4% paraformaldehyde for 15 mins. 0.2% triton X-100 were utilized to increase the permeability of the cell membrane for 15 mins. Following a 1-hour blocking step with 10% donkey serum, sections were incubated with primary antibodies overnight at 4°C, with secondary antibodies at 37°C for 1 hour. The nuclei were stained by DAPI (G1012, Servicebio, Wuhan, China). Representative figures were captured by confocal microscope (Dragonfly/CR-DFLY-201-40, ANDOR, NI, UK).

### IHC staining.

The 3 μm paraffin sections were initially immersed in xylol overnight. Subsequently, they were dewaxed with 2 times of xylol, 2 times of anhydrous ethanol, 95% ethanol, and 75% ethanol. 3% Hydrogen peroxide methanol was used to consume endogenous peroxidase. Antigen retrieval was performed using citrate antigen unmasking solution with microwave methods. Following a 1-hour blocking step with 10% donkey serum, sections were incubated with primary antibodies overnight at 4°C, with secondary antibodies at 37°C for 1 hour, and ABC-AP Kit (Vectorlabs, CA, USA) for 30 minutes. Finally, sections were stained by AEC Kit (Vectorlabs, CA, USA), and nuclei were stained by Hematoxylin. Stained samples were viewed and pictured under a bright field microscope (Ni-E, Nikon, Tokyo, Japan). IHC analysis was performed as described previously.

### ATP detection.

The measurement of ATP level was conducted by ATP Assay Kit (S0026, Beyotime Biotechnology, Wuhan, China). Tissue samples or cultured cells were lysed at 4°C for 15 mins and centrifugated at 4°C for 15 mins 13,400 xg. The supernatant of centrifugation was collected, and luminescence were detected by ATP Assay Kit on microplate reader (PerkinElmer Multimode, MA, USA). The level of sample ATP were standardized with ATP standard.

### Measurement of citrate synthase activity.

Renal citrate synthase activity detection was conducted according to the instruction manual of Citrate Synthase Activity Assay Kit (MAK-193, Sigma, MO, USA). In brief, 0, 8, 16, 24, 32, and 40 nmole/well of GSH were prepared to generate GSH Standard Curve. Freshly isolated kidney samples were weighted and homogenized in ice cold CS assay buffer. GSH or sample supernatant was added to 96 well plate at a final volume of 50 μl. Reaction Mix which contained 43 μl CS Assay Buffer, 5 μl CS Developer, and 2 μl CS Substrate Mix was added to each well. The plate was measured at the absorbance of 412 nm, and then incubated at 25°C until the absorbance value of sample was very close or greater than the value of the highest standard (40 nmole GSH).

### Measurement of mitochondrial OCR.

HK-2 cells (11.2 thousand/well) were seeded on Seahorse XFe24 cell culture plate. TGF-β1 (10 ng/ml) was incubated for 24 hours after cell adherence. XF assays were conducted following the manufacturer’s instructions, supplemented with oligomycin (1 μM), FCCP (1 μM), and antimycin A plus rotenone (0.5 μM). The mitochondrial OCR was measured with Seahorse XFe24 Flux Analyzer (Agilent, CA, USA).

### Oil red O staining.

The cells cultured on coverslips were subjected to Oil Red staining following PBS washing, fixation with a 4% PFA solution, and subsequent staining for 10 minutes using the Oil-red O working solution (O0625-25G, Sigma, MO, USA) in order to visualize lipid droplets. Cell nuclei were stained by Hematoxylin (G-clone, Beijing, China). Animal frozen renal tissues were sectioned into 2 μm, fixed by 4% PFA solution for 30 mins, and stained for 15 min with Oil-red O working solution. Then cell nuclei were stained by Hematoxylin.

### Masson-trichrome staining.

The paraffin sections in 3 μm were used to dewax and rehydration. Then sections were soaked in Bouin solution at 65°C for 3 h. After washing, sections were stained by hematoxylin, ponceau staining solution, and aniline blue staining solution. And sections were differentiated with 1% hydrochloric acid alcohol and then treated with TO transparent agent.

### H&E staining.

The steps of dewaxing and rehydration were same with IHC staining. Sections were stained by Hematoxylin for 10 minutes and Eosin for 30 s. And sections were differentiated with 1% hydrochloric acid alcohol and then treated with TO transparent agent.

### Sirius red staining.

6 μm paraffin sections were used. Dewaxing and rehydration were the same as with IHC. Sections were stained by Sirius Red staining solution (G-clone, Beijing, China) at 37°C for 1 h. Then sections were washed by purified water.

### Mitotracker staining.

After washing with PBS, cell coverslips were incubated with mitotracker (M22425, Invitrogen, CA, USA) at 37°C 30 mins. The coverslips were fixed using 4% PFA solution and stained by Actin-Tracker Green (C1033, Beyotime Biotechnology, Wuhan, China). Cell nuclei were stained by DAPI (G1012, Servicebio, Wuhan, China).

### BODIPY staining.

Cell coverslips were incubated with BODIPY working solution (M9850, AbMole, TX, USA) at 37°C for 30 mins. Then coverslips were fixed using 4% PFA solution. Cell nuclei were stained by DAPI (G1012, Servicebio, Wuhan, China). Animal tissues were frozen sectioned into 2 μm, incubated with BODIPY working solution (M9850, AbMole, USA) at 37°C for 60 mins, then fixed using 4% PFA solution. The nuclei were stained by DAPI (G1012, Servicebio, Wuhan, China).

### Biochemical analysis of serum samples.

The clinical parameters of serum such as AST, ALT, BUN, and glucose were measured by automatic biochemical analyzer. Serum creatinine was measured by creatinine assay kit (DICT-500, bioassay systems, CA, USA).

### Co-IP.

The Co-Immunoprecipitation (Co-IP) was performed by CO-IP Kit (P2197, Beyotime Biotechnology, Wuhan, China). In brief, HK-2 cells, which were exposed to TGF-β for 45 minutes, were lysed in IP lysis with 1× protease inhibitor cocktail and 1× PMSF for at least 10 minutes on ice and centrifugated at 13,400 xg 4°C for 15 mins. 20 μl supernatant was collected as input. The rest of the supernatant was incubated with Agarose A+G and 1 μg antibody (primary antibody or Rabbit IgG) at 4°C. The mixture was diluted with pre-cold PBS to 500 μl and rotated overnight. After washing with precooled PBS solution, the pull-down proteins were boiled with loading buffer at 100°C metal bath for later immunoblotting.

### ChIP.

Chromatin immunoprecipitation (ChIP) was performed by ChIP Kit (P2078, Beyotime Biotechnology, Wuhan, China). In brief, Cells were fixed with 1% formaldehyde in the culture medium, followed by 1× Glycine Solution, and then washed with 1× PBS containing 1% PMSF, as per the instruction manual. Cells were collected and resuspended by SDS Lysis buffer, and DNA was sheared using ultrasonic equipment. The DNA fragments were diluted by ChIP dilution buffer. 20 μl of the sample was collected as input. The remaining samples were incubated with Protein A+G Agarose/Salmon Sperm DNA and 3 μg primary antibody at 4°C overnight at rotary table. Subsequently, samples were washed and purified to conduct PCR amplification and DNA gel electrophoresis.

### DNA-pull down.

The HK-2 cells were exposed to 5 ng/mL TGF-β, and nuclear proteinswere extracted from 5×10^7^ cells with Kit (78833, Thermo fisher, CA, USA). To obtain biotin-labeled DNA probe of PRDM16 promoter, PRDM16-luc plasmid, forward primer: bio-TTCTCTGCCCCAACCCCTG, reverse primer: GGTGTCGGCTCGCGGAATC, PrimeSTAR Max Premix (2×) (R045B, Takara, Tokyo, Japan) were used to PCR amplification. And DNA was recycled after gel electrophoresis with agarose gel DNA Recovery Kit (Guangzhou IGE biotechnology ltd, K110-S, China). Then the nuclear proteins were incubated with biotin-labeled DNA probe of PRDM16 promoter or DNA probe without label for 4°C overnight. The next day, streptavidin magnetic beads (21344 Thermo fisher, CA, USA) were added to the tubules to pull down the DNA-protein complex. After washing and elution, the final pull-down proteins were measured by LC/MS-MS. DNA Probe synthesis, pull-down and LC/MS-MS were performed at Fitgene Biotech Co., LTD (Guangzhou, China).

### Lentivirus-mediated gene expression.

The HK-2 cells were cultured in 6 well culture plates at 20%–30% density. Cells were treated with cotransfection reagent A and P, and lentivirus. The multiplicity of infection (MOI) of HK-2 cells was 10. 12 h after transfection, medium was changed. 24 h after transfection, GFP florescence were observed. 72 h after transfection, 2 μg/ml puromycin was added to medium to kill the nontransfected cells.

### siRNA-mediated knockdown.

HK-2 cells were cultured and seeded on 6 well plates with 50%–60% density. Cells were changed to optimem culture medium. 5 μl siRNA was transfected to each well with 5 μl lipo2000 lipofectamine (Invitrogen, CA, USA). Cells were changed to DMEM/F12 medium with 10% FBS for 24 hours.

### RNA-seq analysis.

Total RNA was extracted from HK-2 cells, which were exposed to TGF-β for 24 hours, and total RNA was extracted from the kidney cortex of *PRDM16^fl/fl^* and *Ksp-cre/PRDM16^fl/fl^* mice, which suffered from UIRI surgery and were subjected to RNA-seq analysis performed by MGI platform. In this study, RNA quality assessment was conducted using a NanoDrop 2000 spectrophotometer (NanoDrop Technologies, Wilmington, DE, USA) and a Bioanalyzer 2100 system (Agilent Technologies, CA, USA). Subsequently, mRNA isolated through Oligo(dT)-attached magnetic beads was fragmented using fragment buffer under optimal conditions. The fragments were used to create first-strand cDNA through random hexamer-primed reverse transcription. This was followed by second-strand cDNA synthesis and purification using AMPure XP Beads. The cDNA library was constructed through repair, PCR amplification, and purification. Qubit 2.0 and Agilent 2100 bioanalyzer were used for quantification. Sequencing was performed by BGI Hong Kong on the MGI DNBseq T7 platformas 150 bp paired-end reads, the data of per sample was 6G. Raw reads from RNA-seq libraries undergo filtering to remove reads containing adapters or reads of low quality. After filtering, statistical analysis was performed using R software. The library construction, sequencing, and analysis were performed at Wuhan Generead Biotechnology Co. Ltd (Wuhan, China).

### Transmission electron microscopy.

To observe the structure of mitochondria, HK-2 cells and kidney tissues were fixed with 2.5% glutaraldehyde at 4°C. Then, the samples were washed 15 minutes with 0.1 mol/L PBS for 6 times, and fixed with 1% osmium tetroxide for 1 hour at room temperature. After fixation, samples were dehydrated with 3% acetone for 15 minutes, 5% acetone for 15 minutes, 70% acetone for 15 minutes, 90% acetone for 15 minutes, 100% acetone for 10 minutes for 4 times in series. And next, ethoxyline resin was used to embed samples for 12 hours at 37°C. Finally, we can observe the samples by transmission electron microscope after section.

### Statistics.

All data with individual points are expressed as mean ± SEM. *P* < 0.05 was considered significant. Before downstream analysis, normal distribution and equal variance tests were performed. Two-tailed Student’s unpaired *t* test analysis was utilized to compare between 2 groups. One-way ANOVA followed by Tukey’s post test was performed to compare more than 3 groups. Spearman correlation analysis was used to assess the relationship between the ratio of PRDM16 positive–area and other variables.

### Study approval.

The experiments involving animals were carried out in compliance with the ethical guidelines and protocols approved by the ethics committee of Tongji Medical College, Huazhong University of Science and Technology, China (Approval no. 2797). The investigation of human biopsy samples was approved by the Medical Ethics Committee of Union Hospital, Tongji Medical College, Huazhong University of Science and Technology, China (Approval no. UHCT230185).

### Data availability.

All data used in this study are available in this article. The Supporting Data Values file includes all individual data points for all figures can be found in supplementary files. The raw data of RNA-sequencing was deposited at GEO and had been made public with the accession numbers GSE240749 and GSE240748. The proteomics data of DNA pull down were deposited at PRIDE database with accession number PXD044753.

## Author contributions

QY and CZ designed the study; QY, BT, and HS collected and analyzed the clinical data; QY, BT, YZ, CW, Yaru Xie, and Yjuan Xie performed animal models; QY, BT, YZ, and Yaru Xie performed in vitro experiments; QY and BT prepared figures and tables; QY and BT wrote the paper; YL and CZ revised and approved the final version of manuscript.

## Supplementary Material

Supplemental data

Unedited blot and gel images

Supporting data values

## Version 1 08/04/2025

In-Press Preview

## Version 2 09/09/2025

Electronic publication
