# Supplementary material for: PRDM16 acts as a therapeutic downstream target of TGF-β signaling in chronic kidney disease
Source: JCI Insight. 2025 Jul 29;10(17):e191458. doi: 10.1172/jci.insight.191458 (PMC12487692; doi:10.1172/jci.insight.191458)
Supplement: Supplemental data [file jciinsight-10-191458-s164.pdf]

Supplementary Information

PRDM16 acts as a therapeutic downstream target of TGF-β signaling in chronic kidney disease

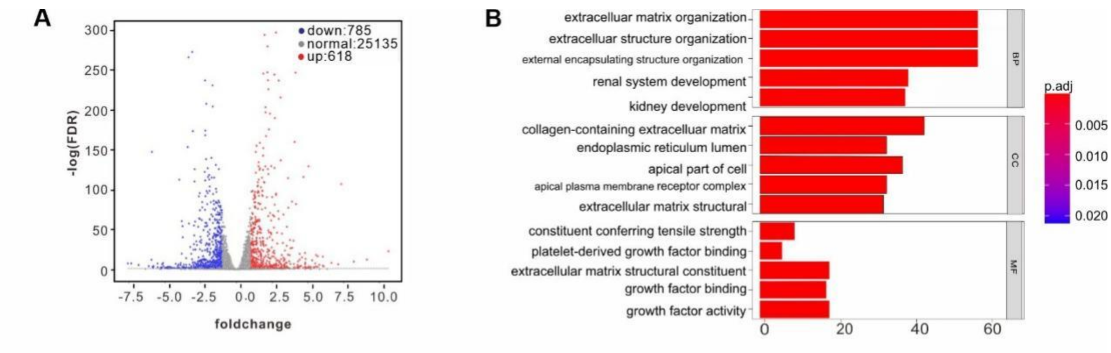

Fig. S1. The bioinformatics analysis of RNA sequencing.

(A) Volcano plot of RNA-sequencing. HK-2 cells were treated with TGF-β for 24 hours. The total RNA was extracted and conducted RNA sequence; (B) GO analysis of RNA-sequencing showed that the expression of the extracellular matrix changed a lot after TGF-β treatment.

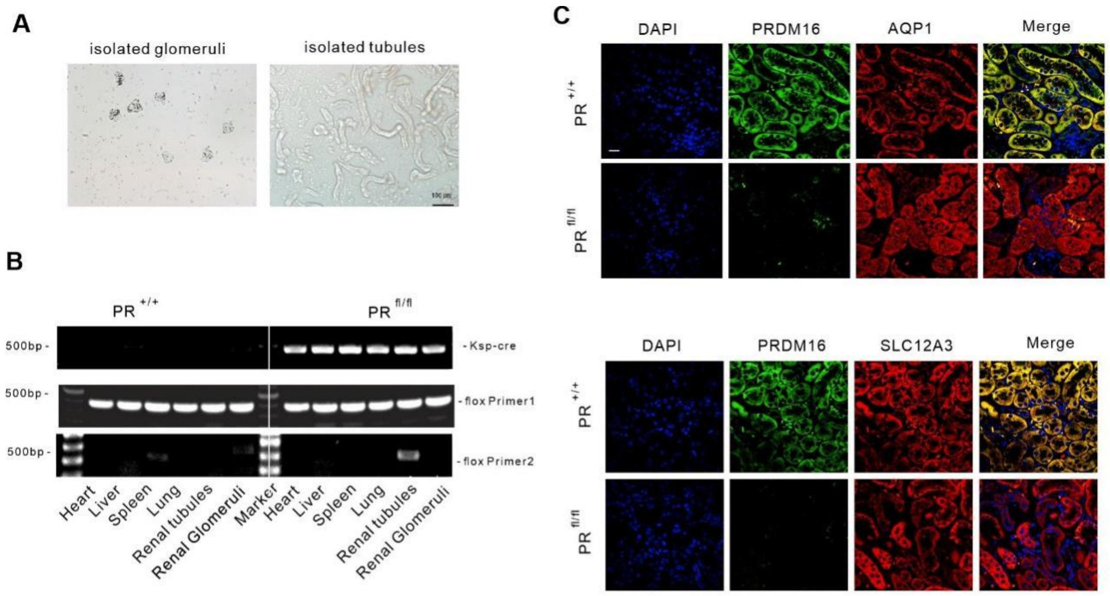

Fig. S2. *Prdm16* was deleted in the tubules of tubular-specific knockout *Prdm16* mice.

(A) Glomeruli and tubules were isolated from transgenic mice; (B) PCR results of mice genotyping with genomic DNA isolated from heart, liver, spleen, lung, glomeruli, and tubules; (C) Immunofluorescence co-staining of PRDM16 with proximal tubule marker AQP1 and distal tubule marker SLC12A3, respectively. Scale Bar = 20μm.

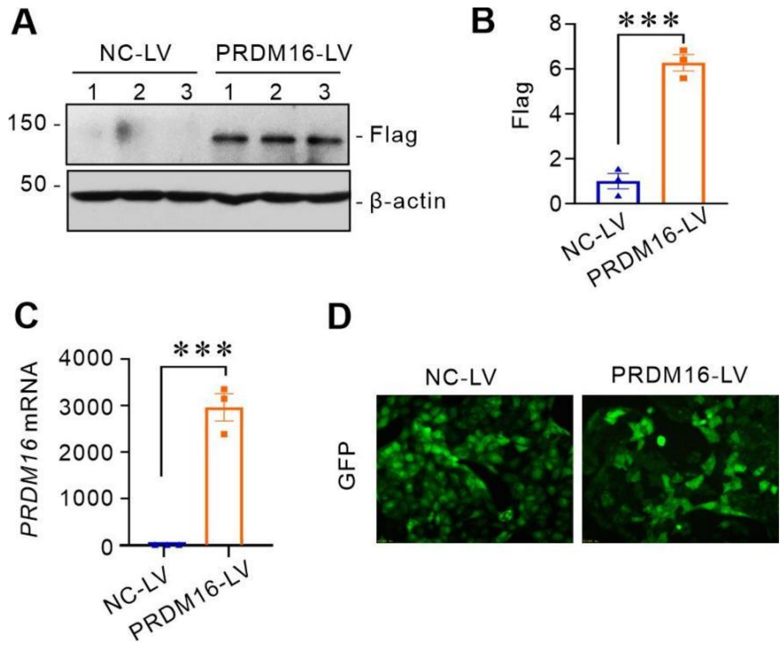

Fig. S3. Identification of PRDM16 stably overexpression HK-2 cell line.

(A-B) Representative Western blotting (A) (n = 3 blots in total) and quantification of Flag (B) in HK-2 cells transfected with PRDM16 lentivirus (PRDM16-LV) and negative control virus (NC-LV); (C) Relative mRNA level of *PRDM16* (n=3); (D) Representative images of the expression of GFP. Data are mean ± SEM. ***$p < 0.001$, Two-tailed Student's unpaired t-test analysis (B, C).

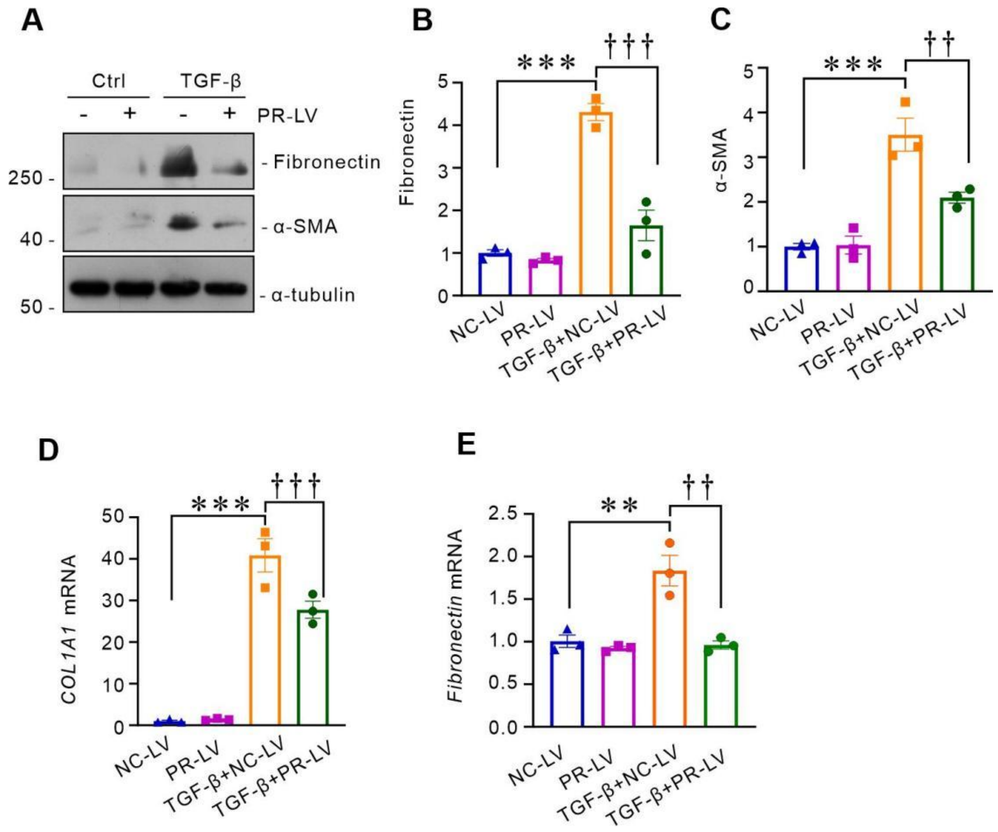

Fig. S4. PRDM16 inhibited partial EMT of tubular epithelial cells.

(A-C) Stably transfected HK-2 cells were treated with 5ng/ml TGF-β for 24 hours. Representative Western blotting (A) (n = 3 blots in total) and quantification of Fibronectin (B) and α- SMA (C); (D-E) Relative mRNA level of *COL1A1* and *Fibronectin* in stably transfected HK-2 cells treated with 5ng/ml TGF-β (n=3) for 24 hours. Data are mean ± SEM. **$p < 0.01$, ***$p < 0.001$; †$p < 0.05$, ††$p < 0.01$, †††$p < 0.001$; One-way ANOVA followed by Tukey's post-test (B-E).

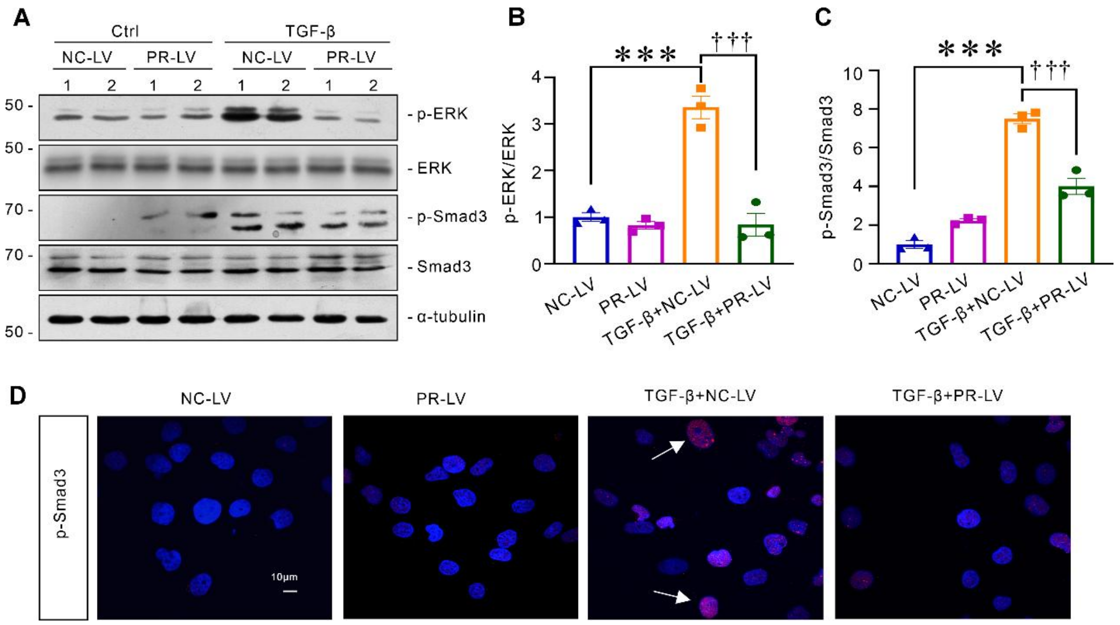

Fig. S5 PRDM16 overexpression inhibits TGF-β signaling

(A-C) Stably transfected HK-2 cells were harvested after 5ng/ml TGF-β treatment for 45 minutes. Representative Western blotting (A) (n = 3) and quantification of phospho-ERK (p-ERK/ERK) (B) and phospho-Smad3 (p-Smad3/Smad3) (C) levels; (D) Representative images of p-Smad3 immunofluorescence in cells exposed to 5 ng/ml TGF-β for 45 minutes. Scale bar = 10 μm. White arrow: positive staining. Data are mean ± SEM. ***$p < 0.001$;†††$p < 0.001$.One-way ANOVA followed by Tukey's post-test (B-C).

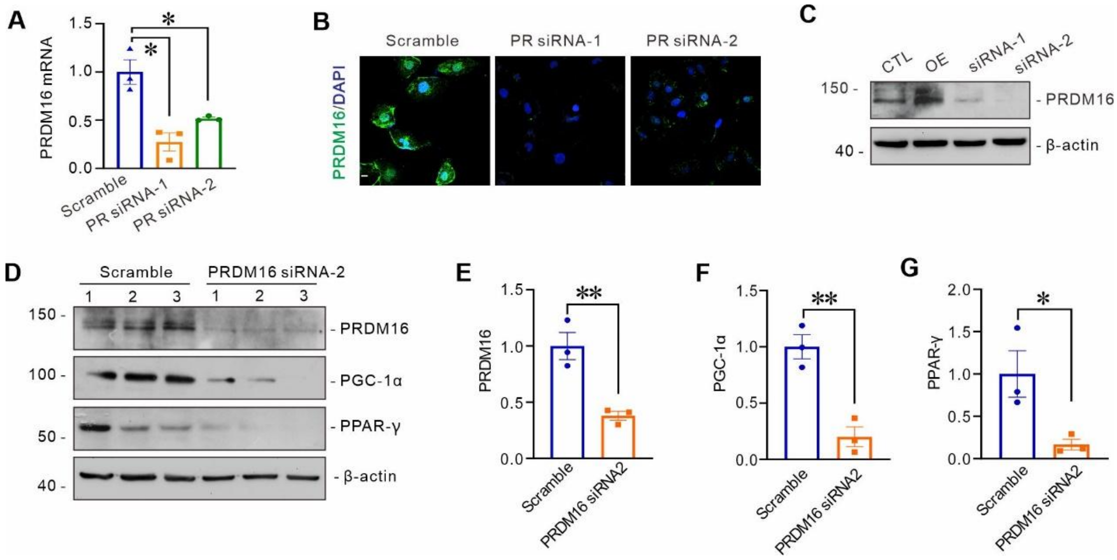

Fig. S6. PRDM16 knockdown decreased the expression of PGC-1α and PPAR-γ.

(A) HK-2 cells were transfected with PRDM16 siRNA1 (PR siRNA-1), PRDM16 siRNA2 (PR siRNA-2) or Scramble for 24 hours. Relative mRNA level of *PRDM16*; (B) Representative images of immunofluorescence staining of PRDM16. Scale Bar = 10μm; (C) Representative Western blotting of PRDM16 in HK-2 cells which were treated with PRDM16 overexpression lentivirus, and two siRNAs. (D-G) Representative Western blotting (D) (n = 3 blots in total) and quantification of PRDM16 (E), PGC-1α (F), and PPAR-γ (G). *p < 0.05, **p < 0.01, Two-tailed Student's unpaired t-test analysis (A, E-G).

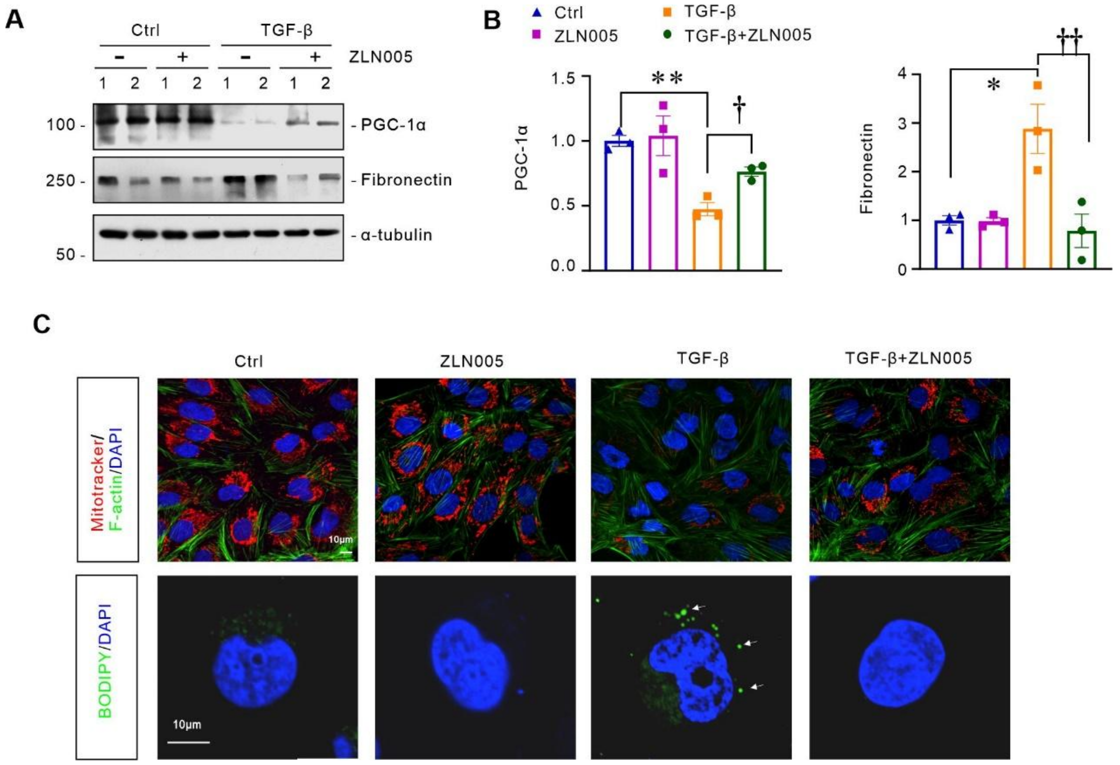

Fig. S7. PGC-1α activator, ZLN005 inhibited TGF-β induced mitochondrial injury. (A-B) HK-2 cells were treated with 5ng/ml TGF-β and 5μmol/L ZLN005 for 24 hours. Representative Western blotting (A) (n = 3 blots in total) and quantification (B) of PGC-1α and Fibronectin; (C) Representative images of mitotracker staining and BODIPY Staining in HK-2 cells treated with 5ng/ml TGF-β and ZLN005. Scale Bar = 10μm. White Arrow pointed to positive lipid drops. Data are mean ± SEM. $*p < 0.05$, $**p < 0.01$; $†p < 0.05$, $††p < 0.01$; One-way ANOVA followed by Tukey's post-test (B).

Table S1 Clinical data, and the ratio of PRDM16 and PGC-1α positive area of control and IgA patients

Number	Stage	Age	sex	BUN (mmol/L)	Scr(umol/L)	CysC (mg/L)	eGFR (ml/ (min/1.73^2))	UPE (mg/24h)	PRDM16 (%)	PGC-1α (%)
1	T2	47	male	12.5	309.5	2.7	19.61	6299	7.123	8.983
2	T2	50	male	/	/	/	/	/	2.039	5.157
3	T2	52	female	5.7	120	1.61	44.51	6920	4.67	6.746
4	T2	52	male	4.6	108.3	1.34	67.4	3710	9.016	10.033
5	T1	48	female	9.3	124.4	1.56	44.18	1301	7.252	2.975
6	T1	44	male	5.2	145.9	1.93	49.73	1514	13.297	11.264
7	T1	44	female	5.7	83.9	1.36	73.16	1406	5.268	2.871
8	T1	47	female	6.92	79.5	0.78	76.46	1336	6.841	9.304
9	T0	48	male	4.4	76.5	1.02	102.28	265	15.099	5.197
10	T0	48	female	4.3	69.9	0.64	88.7	966	21.304	18.126
11	T0	44	female	3.1	75.4	1.19	83.25	882	11.304	2.971
12	T0	44	male	3.71	84.7	1.07	95.97	4165	9.696	3.081
13	control	64	female	3.67	79.6	1.44	72.59	/	31.741	33.852
14	control	62	female	4.03	54.6	1.43	58.65	/	26.012	29.187
15	control	47	female	3.75	68.9	0.78	115.98	/	26.58	25.368

Table S2 Primers for real-time RT-PCR.

Species	gene	Forward (5' to 3')	Reverse (5' to 3')
Mouse	*β-actin*	CAGCTGAGAGGGAAATCGTG	CGTTGCCAATAGTGATGACC
Mouse	*PRDM16*	CAGCACGGTGAAGCCATTC	GCGTGCATCCGCTTGTG
Mouse	*Ppargc1a*	CACCAAACCCACAGAAAACAG	GGGTCAGAGGAAGAGATAAAGTTG
Mouse	*Vimentin*	GATCGATGTGGACGTTTCCAA	ATACTGCTGGCGCACATCAC
Mouse	*Fibronectin*	GATGAGCTTCCCCAACTGGT	CTGGGTTGTTGGTGGGATGT
Mouse	*Col1a1*	ATCTCCTGGTGCTGATGGAC	ACCTTGTTTGCCAGGTTCAC
Mouse	*Acox2*	ATAACCGAGTCGTTCTGCCAAT	TTTCAGAGCATTGGCCATAGAA
Mouse	*Col3a*	ACAGCTGGTGAACCTGGAAG	ACCAGGAGATCCATCTCGAC
Mouse	*Tfam*	GAGCAGCTAACTCCAAGTCAG	GAGCCGAATCATCCTTTGCCT
Mouse	*Cpt1a*	GGTCTTCTCGGGTCGAAAGC	TCCTCCCACCAGTCACTCAC
Mouse	*H-Ras*	AGGAAGGAAAGAGGCGGGAAGG	CACAGGAGCAAGGCAGATGATGG
Mouse	*Ksp-cre*	GCAGATCTGGCTCTCCAAAG	AGGCAAATTTTGGTGTACGG
Mouse	*Floxp primer1*	CCCACAGTGACACACCCTAC	GAGATCACGAGGAACCCAC
Mouse	*Floxp primer2*	CCCACAGTGACACACCCTAC	ATTCCACCCTACCAGGACCA
Human	*β-actin*	CTCACCATGGATGATGATATCGC	AGGAATCCTTCTGACCCATGC
Human	*PRDM16*	CGAGGCCCCTGTCTACATTC	GCTCCCATCCGAAGTCTGTC
Human	*PPARGC1A*	AAAGGATGCGCTCTCGTTCA	GGAATATGGTGATCGGGAACA

Human	*Fibronectin*	AGCAAGCCCGGTTGTTATGA	CCCACTCGGTAAGTGTTCCC
Human	*TFAM*	CGCTCCCCCTTCAGTTTTGT	CCAACGCTGGGCAATTCTTC
Human	*COL1A1*	GAGGGCCAAGACGAAGACATC	CAGATCACGTCATCGCACAAC
Human	*ACOX2*	TCTTCTACCAACCAGCCCTG	CTCTCTATGTCGGGGTGCAT
Human	*H-Ras*	CGGAAGCAGGTGGTCATTGATGG	GCAGCCAGGTCACACTTGTTCC
Human	ChIP Primer1	TTCTCTAGGCGACACTCACC	TGCTCAGATTCGAACCCAGA
Human	ChIP Primer2	GGTGGGTAAAGGGGTCGATA	TCCCTGCTCAGATTCGAACC
Human	ChIP Primer3	ATGTGCGAAGGTGTCCAAAC	GCGGAATCTCTCTCCTCCTC

Table S3 Key resources table

REAGENT or RESOURCE	SOURCE	IDENTIFIER
Antibodies		
Rabbit polyclonal anti-PRDM16(1:1000 For WB; 1:50 For IHC)	Abcam	Cat#ab106410
Sheep polyclonal anti-PRDM16(1:1000 For WB; 1:50 For IHC)	R&D system	AF6295
Rabbit polyclonal anti-AQP1 (1:200 For IF)	ProteinTech	20333-1-AP
Rabbit polyclonal anti-SLC12A3 (1:200 For IF)	Abcam	ab95302
Rabbit monoclonal anti-PGC-1α (1:1000 For WB; 1:50 For IHC)	Boster	Cat#BM4898
Rabbit polyclonal anti-α- SMA (1:1000 For WB; 1:50 For IHC)	ProteinTech	Cat#14395-1-

		AP
Rabbit polyclonal anti-Fibronectin (1:10000 For WB; 1:100 For IHC and IF)	Sigma	Cat#F3648
Rabbit polyclonal anti-H-RAS (1:1000 For WB)	ProteinTech	Cat#18295-1-AP
Rabbit polyclonal anti-Phospho-Smad3(1:1000 For WB)	Santa Cruz	Cat#sc-130218
Rabbit monoclonal anti-Phospho-Smad3(1:1000 For WB; 1:50 For IHC,1ug for Co-IP, 3ug for ChIP)	CST	Cat#9520T
Mouse monoclonal anti-FLAG (1:1000 For WB)	Sigma	Cat#F1804
Rabbit monoclonal anti-PPAR-γ (1:1000 For WBC)	CST	Cat#2435s
Rabbit polyclonal anti-CPT1A (1:2000 For WB)	ProteinTech	Cat#15184-1-AP
Rabbit polyclonal anti-GAPDH (1:10000 For WB)	ProteinTech	Cat No.60004-1-Ig
Mouse monoclonal anti-β-Actin(1:10000 For WB)	Santa Cruz	Cat#sc-47778
HRP-conjugated mouse monoclonal anti- α-Tubulin （1:10000 For WB）	ProteinTech	Cat No.HRP-66031
Donkey polyclonal Secondary Antibody to Rabbit IgG - H&L (Alexa Fluor® 594-AffiniPure)	Jackson	Cat#711-585-152
Biotin-SP (long spacer) AffiniPure Donkey Anti-Rabbit IgG (H+L)	Jackson	Cat#711-065-152
Rabbit IgG	Beyotime	Cat#A7016

	Biotechnology	
Chemicals, Peptides, and Recombinant Proteins		
ProDots Recombinant Human TGF-beta 1 Protein	RnD	Cat#PRD240-01
SIS3	MCE	Cat#HY-13013
ZLN005	MCE	Cat#HY-17538
Oil Red O	Sigma-Aldrich	Cat# O0625
mitotracker	Invitrogen	Cat#M22425
Bodipy	AbMole	Cat#M9850
Phosphatase Inhibitor Cocktail	Servicebio	Cat#G2006
100mM PMSF	Beyotime Biotechnology	Cat# ST506-2
Lipofectamine 2000 transfection reagent	Invitrogen	Cat# 11668500
Actin-Tracker Green	Beyotime Biotechnology	Cat#C1033
DAPI	Servicebio	Cat#G1012
Critical Commercial Assays		
Immobilon Western Chemiluminescent HRP substrate	Applygen	Cat#P1010
Co-IP Kit	Beyotime Biotechnology	Cat#P2197
ChIP Kit	Beyotime Biotechnology	Cat#P2078

creatine assay kit	bioassay systems	Cat#DICT-500
Deposited Data		
RNA-sequencing data	This paper	N/A
Experimental Models: Cell Lines		
Homo Sapiens：HK-2	ATCC	N/A
Experimental Models: Organisms/Strains		
Mouse: PRDM16 flox/flox (C57BL/6J;129)	Shanghai Southern Model Biotechnology Development Co., Ltd.	N/A
Mouse: C57BL/6J;129	Vital River Laboratory Animal Technology Co. Ltd.	N/A
Mouse: PRDM16$^{+/+}$and PRDM16 $^{fl/fl}$ (C57BL/6J;129)	bred by Shulaibao (Wuhan) Biotechnology Co., Ltd	N/A
Mouse: Ksp-cre	Cyagen Biosciences Co. Ltd.	N/A

PRDM16 siRNA1	RIBOBIO	stB0012946A
PRDM16 siRNA2	RIBOBIO	SiG000063976B
H-Ras siRNA1	RIBOBIO	stB0003623A
H-Ras siRNA2	RIBOBIO	stB0003623B
NC-siRNA	RIBOBIO	SiN0000001-1-5
Recombinant DNA		
GV703-PRDM16	Shanghai Genechem Co.,Ltd.	N/A
GV141-PRDM16	Shanghai Genechem Co.,Ltd.	N/A
GV238-PRDM16-luc	Shanghai Genechem Co.,Ltd.	N/A
Software and Algorithms		
ImageJ	National Institutes of Health	https://imagej.nih.gov/ij/
GraphPad Prism 8.0	GraphPad	https://www.graphpad.com/

Corel Draw X8	Corel Draw	https://www.corel.com/cn/
Adobe Photoshop CS6	Adobe	https://www.adobe.com/cn
