# Supplementary material for: PRDM16 acts as a therapeutic downstream target of TGF-β signaling in chronic kidney disease
Source: JCI Insight. 2025 Jul 29;10(17):e191458. doi: 10.1172/jci.insight.191458 (PMC12487692; doi:10.1172/jci.insight.191458)

fig 1F

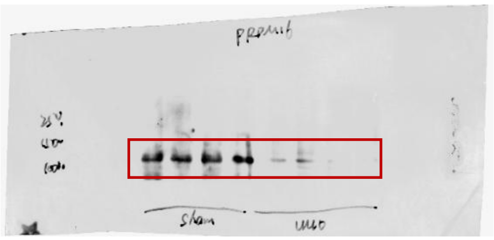

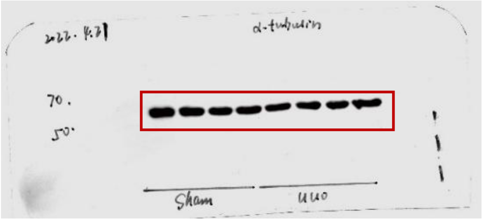

Fig 1I

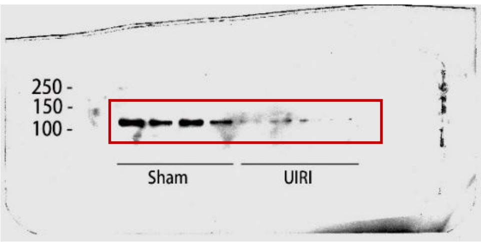

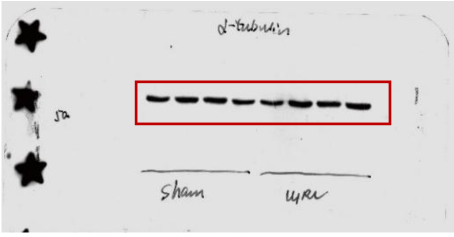

Fig 2A

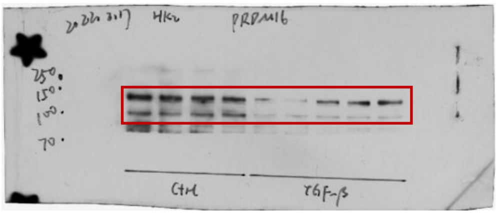

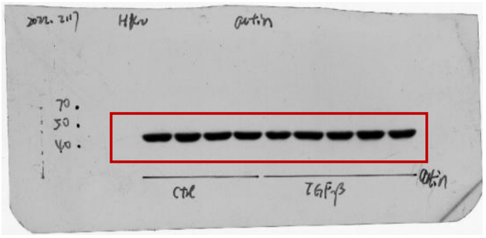

Fig 2D

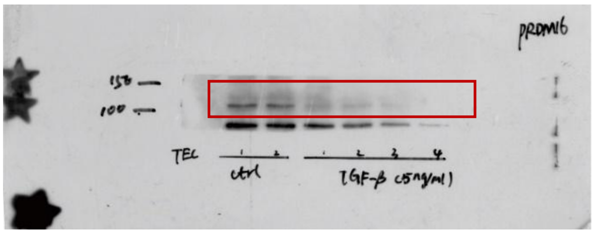

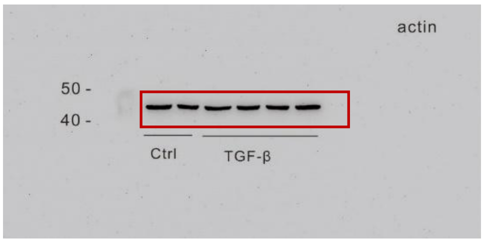

Fig 2G

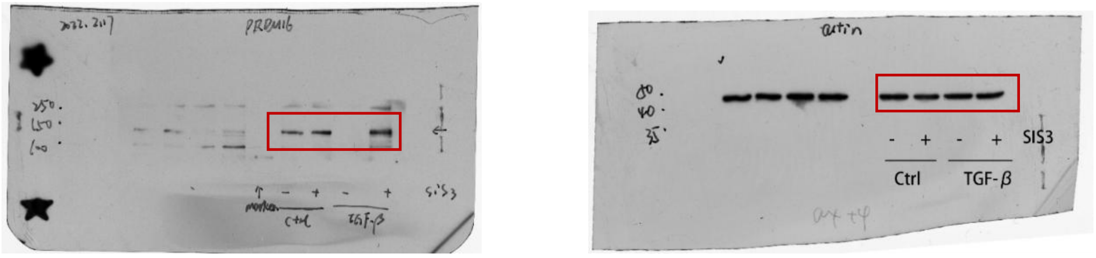

Fig 2P

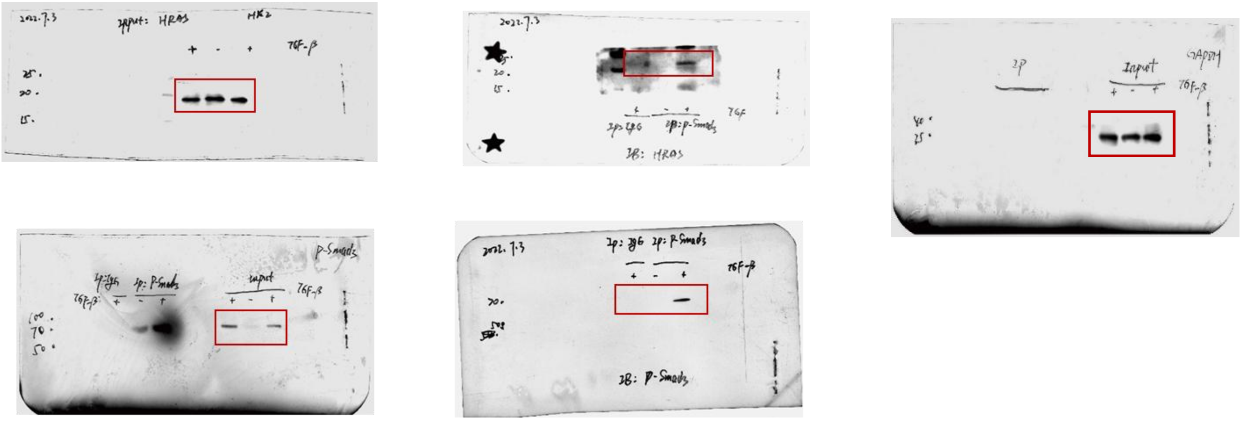

Fig 2R

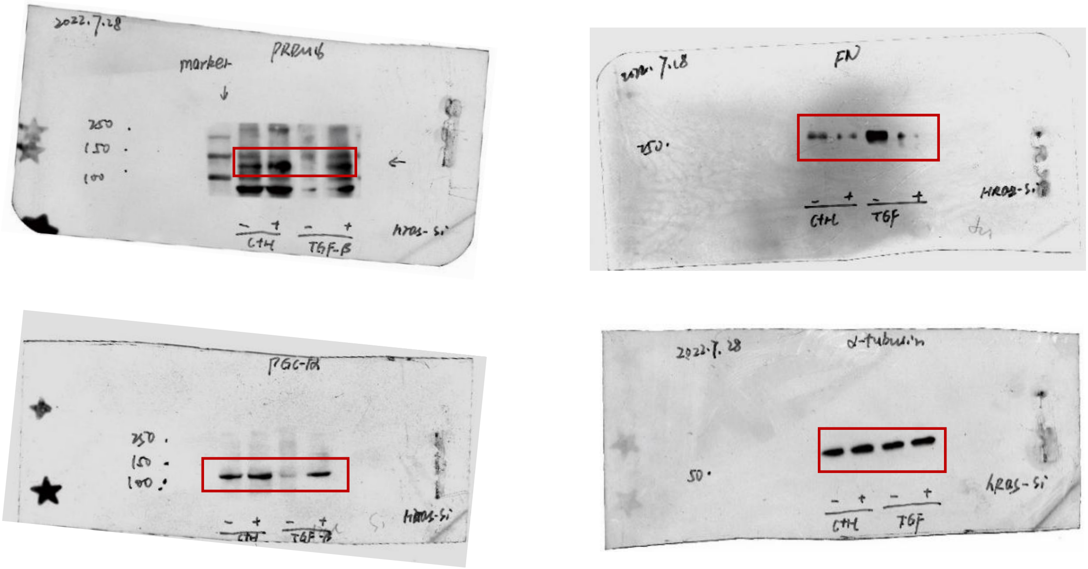

Fig 2T

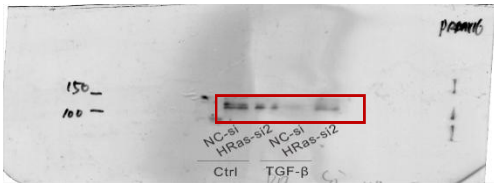
150
100
NC-si
HRas-si2
NC-si
HRas-si2
Ctrl
TGF-β

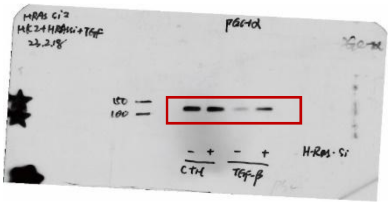
150
100
- + - +
H-Ras-si

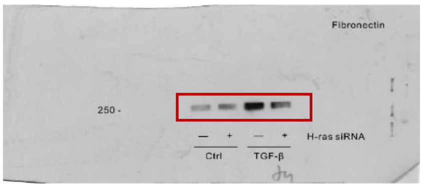
Fibronectin
250 -
— + — +
H-ras siRNA
Ctrl
TGF-β

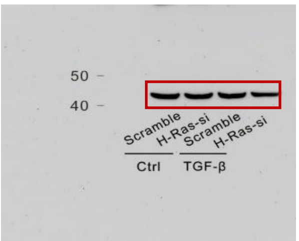
50 -
40 -
Scramble
H-Ras-si
Scramble
H-Ras-si
Ctrl
TGF-β

Fig 3J

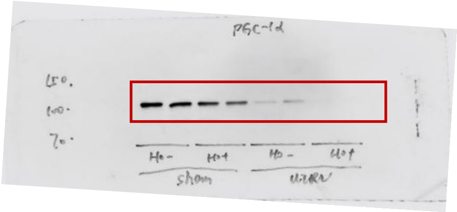

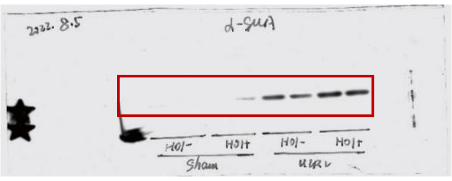

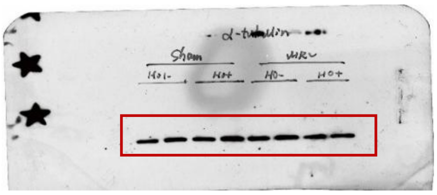

Fig 4F

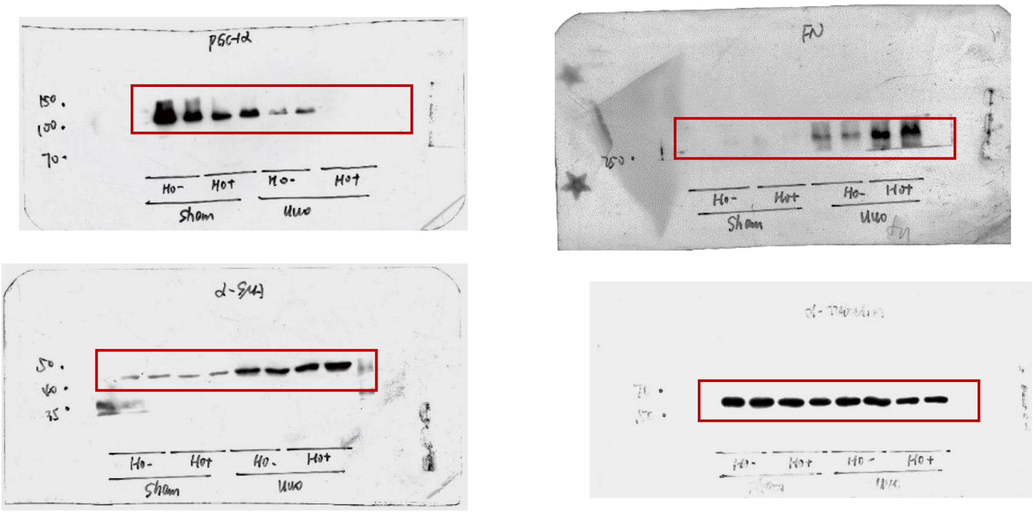

Fig 5B

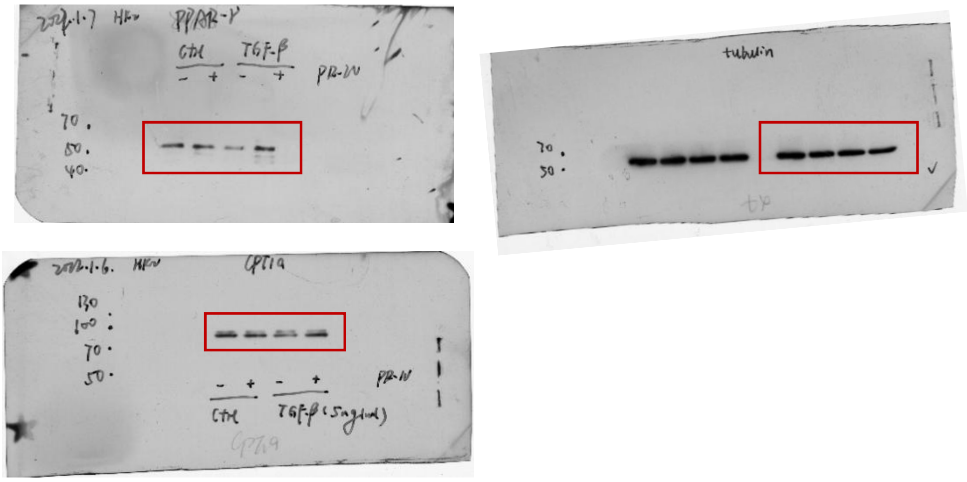

Fig 6C

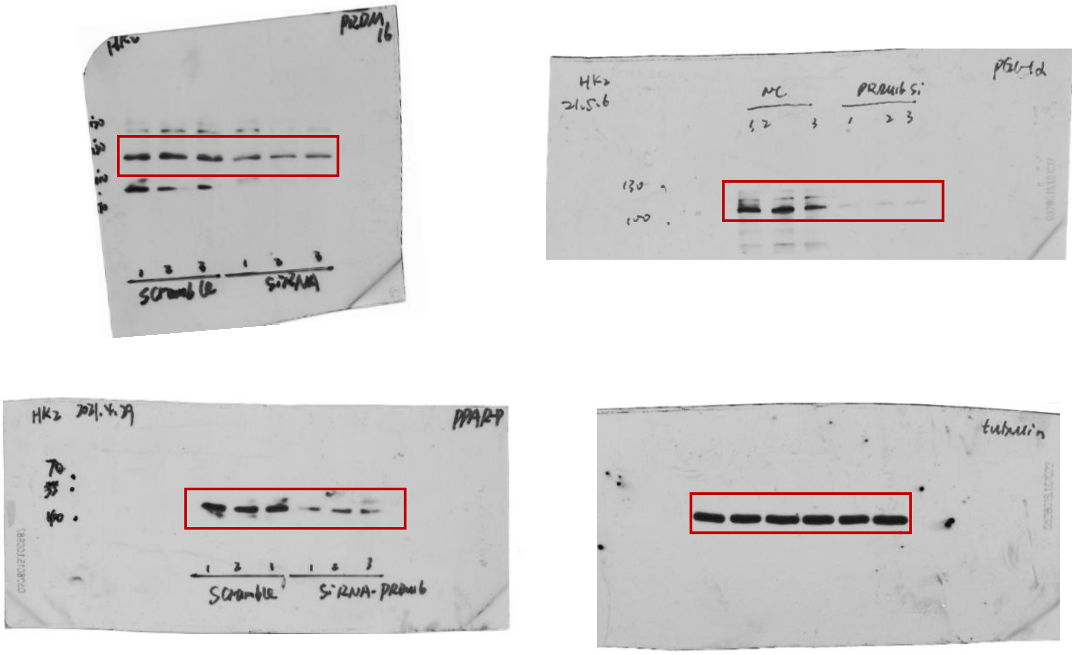

Fig 6G

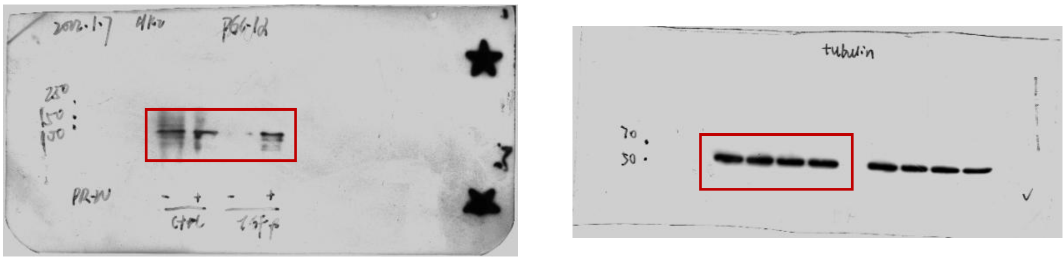

Fig 6J

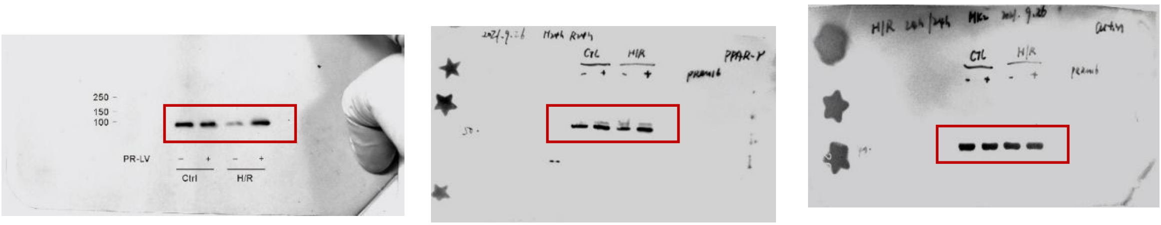

Fig 6M

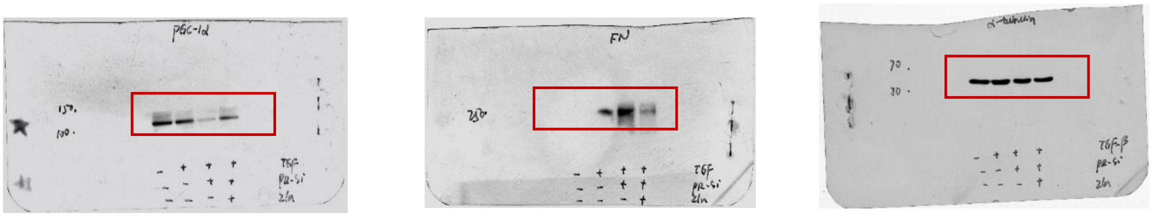
FN
250
70
30
TGF-β
PR-si

Fig 7M

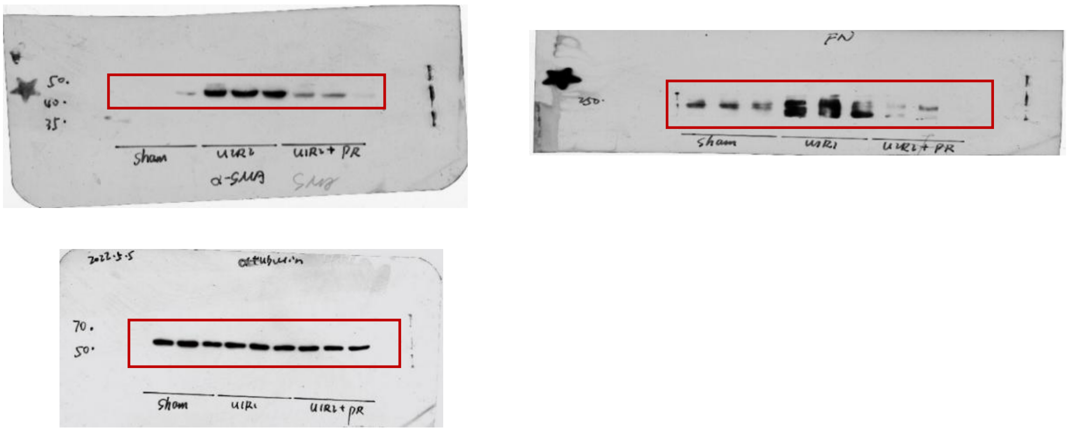
50
40
35
Sham
UIRI
UIRI+PR
α-SMA
FN
250
70
50

Fig 8B

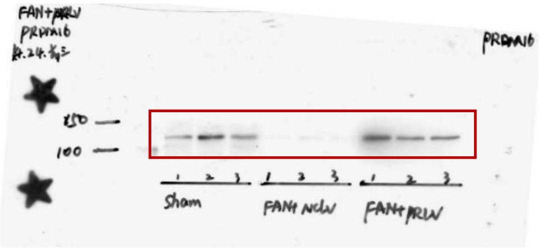

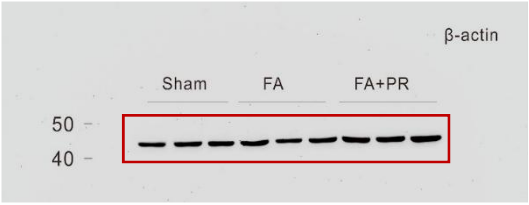

Fig 8O

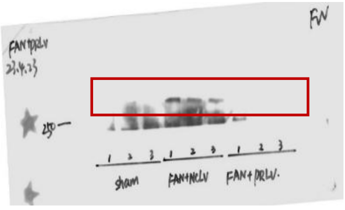

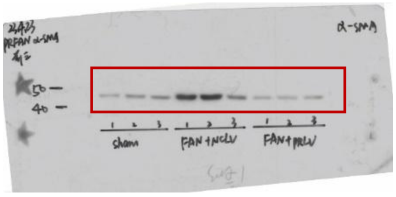

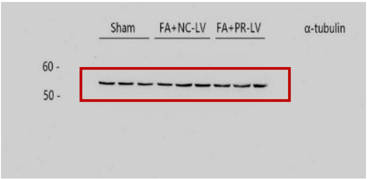

Fig S3A

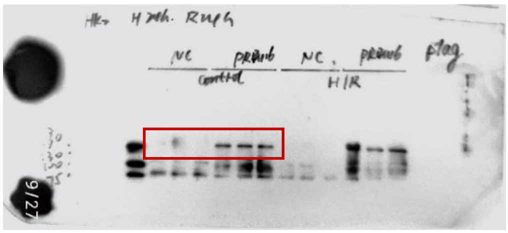

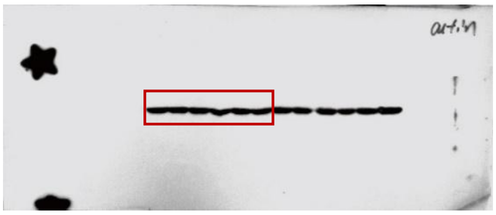

Fig S4A

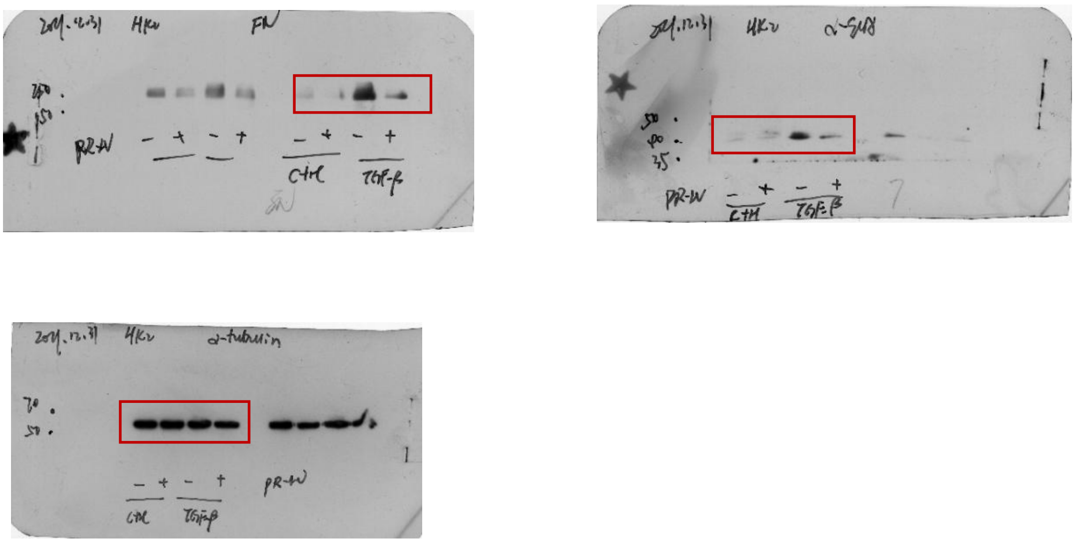

Fig S5A

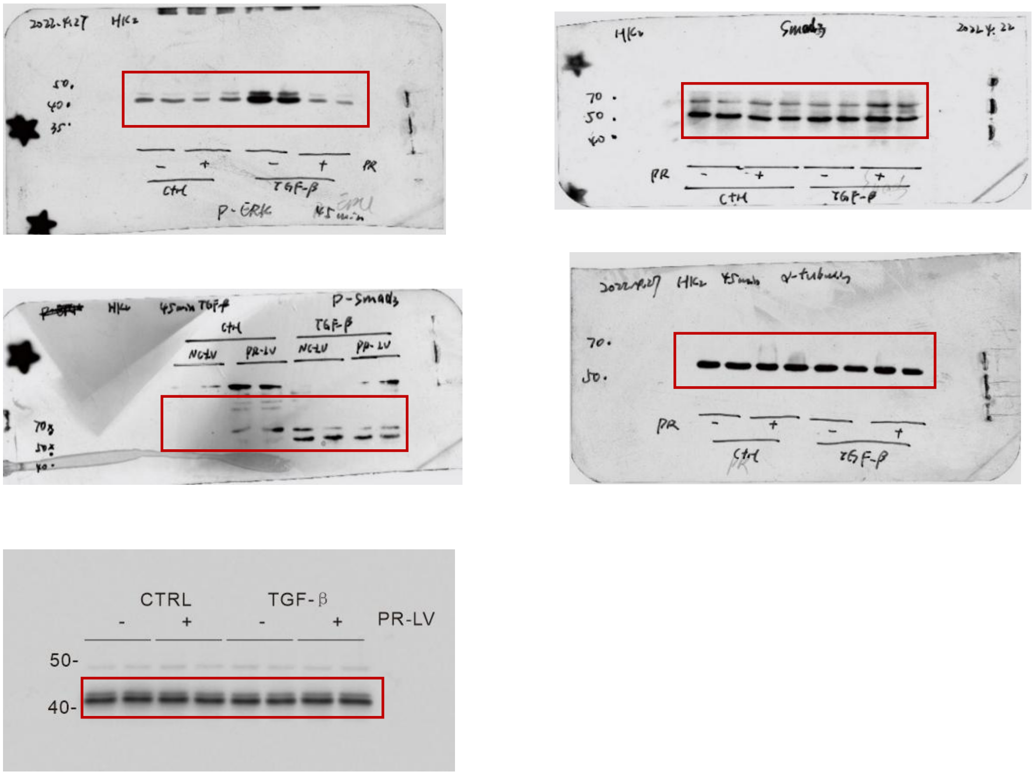

Fig S6C

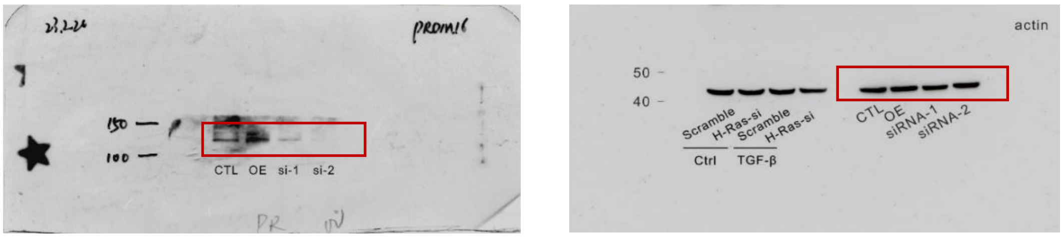

Fig S6D

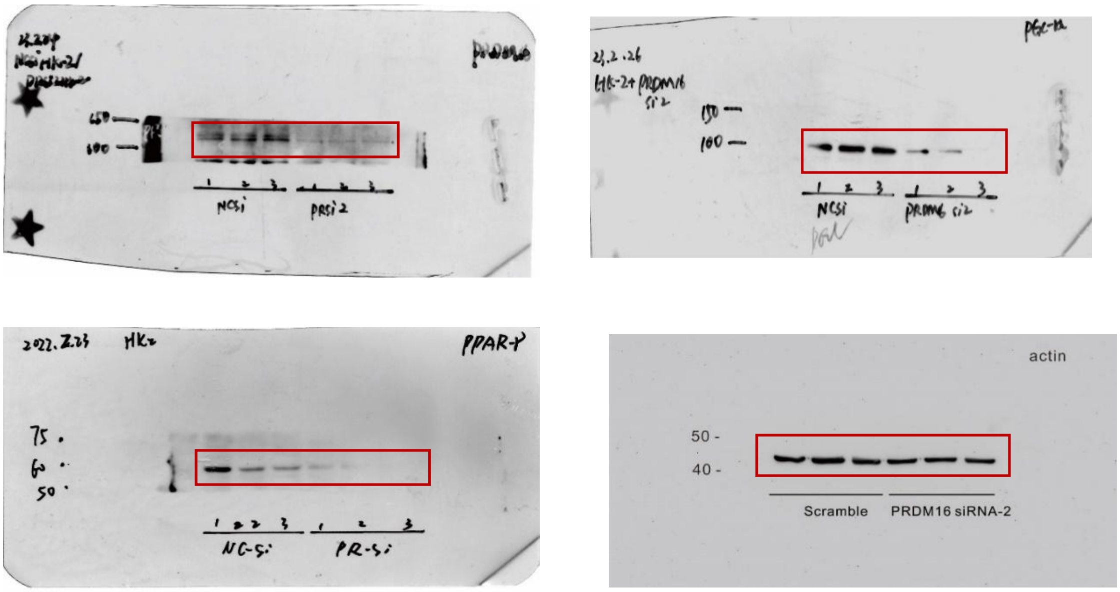

Fig S7A

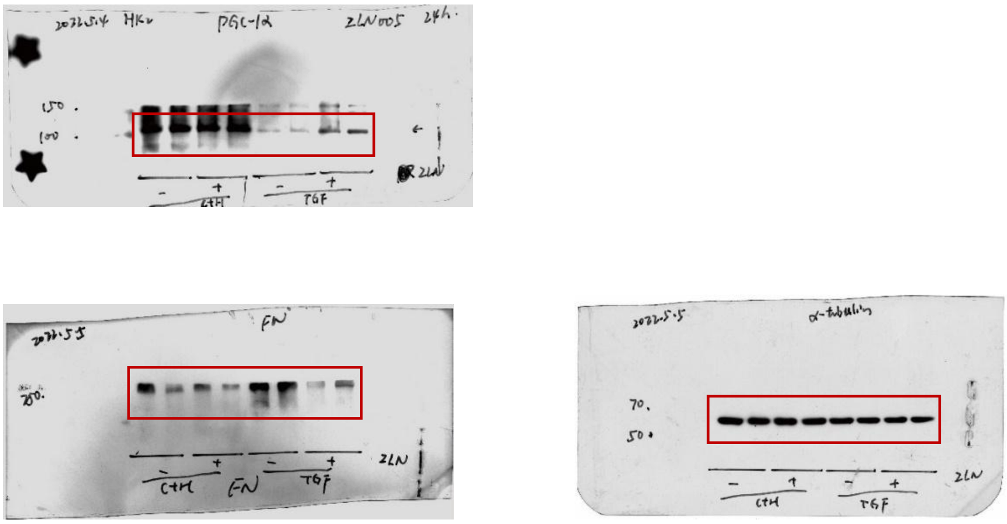

Supplement: Unedited blot and gel images [file jciinsight-10-191458-s165.pdf]
